# Evidence-based gene models for structural and functional annotations of the oil palm genome

**DOI:** 10.1186/s13062-017-0191-4

**Published:** 2017-09-08

**Authors:** Kuang-Lim Chan, Tatiana V. Tatarinova, Rozana Rosli, Nadzirah Amiruddin, Norazah Azizi, Mohd Amin Ab Halim, Nik Shazana Nik Mohd Sanusi, Nagappan Jayanthi, Petr Ponomarenko, Martin Triska, Victor Solovyev, Mohd Firdaus-Raih, Ravigadevi Sambanthamurthi, Denis Murphy, Eng-Ti Leslie Low

**Affiliations:** 1 Advanced Biotechnology and Breeding Centre, Malaysian Palm Oil Board, No. 6, Persiaran Institusi, Bandar Baru Bangi, 43000 Kajang, Selangor, Malaysia; 2 Faculty of Science and Technology, Universiti Kebangsaan Malaysia, 43600 Bangi, Selangor Malaysia; 3 Department of Biology, University of La Verne, La Verne, California, 91750 USA; 4 Spatial Sciences Institute, University of Southern California, Los Angeles, CA 90089 USA; 5 Genomics and Computational Biology Research Group, University of South Wales, Pontypridd, CF371DL UK; 6 Children’s Hospital Los Angeles, University of Southern California, Los Angeles, CA 90089 USA; 7 Softberry Inc., 116 Radio Circle, Suite 400, Mount Kisco, NY 10549 USA

**Keywords:** Oil palm, Gene prediction, Seqping, Fatty acids, Intronless, Resistance genes

## Abstract

**Background:**

Oil palm is an important source of edible oil. The importance of the crop, as well as its long breeding cycle (10-12 years) has led to the sequencing of its genome in 2013 to pave the way for genomics-guided breeding. Nevertheless, the first set of gene predictions, although useful, had many fragmented genes. Classification and characterization of genes associated with traits of interest, such as those for fatty acid biosynthesis and disease resistance, were also limited. Lipid-, especially fatty acid (FA)-related genes are of particular interest for the oil palm as they specify oil yields and quality. This paper presents the characterization of the oil palm genome using different gene prediction methods and comparative genomics analysis, identification of FA biosynthesis and disease resistance genes, and the development of an annotation database and bioinformatics tools.

**Results:**

Using two independent gene-prediction pipelines, Fgenesh++ and Seqping, 26,059 oil palm genes with transcriptome and RefSeq support were identified from the oil palm genome. These coding regions of the genome have a characteristic broad distribution of GC_3_ (fraction of cytosine and guanine in the third position of a codon) with over half the GC_3_-rich genes (GC_3_ ≥ 0.75286) being intronless. In comparison, only one-seventh of the oil palm genes identified are intronless. Using comparative genomics analysis, characterization of conserved domains and active sites, and expression analysis, 42 key genes involved in FA biosynthesis in oil palm were identified. For three of them, namely *EgFABF, EgFABH* and *EgFAD3*, segmental duplication events were detected. Our analysis also identified 210 candidate resistance genes in six classes, grouped by their protein domain structures.

**Conclusions:**

We present an accurate and comprehensive annotation of the oil palm genome, focusing on analysis of important categories of genes (GC_3_-rich and intronless), as well as those associated with important functions, such as FA biosynthesis and disease resistance. The study demonstrated the advantages of having an integrated approach to gene prediction and developed a computational framework for combining multiple genome annotations. These results, available in the oil palm annotation database (http://palmxplore.mpob.gov.my), will provide important resources for studies on the genomes of oil palm and related crops.

**Reviewers:**

This article was reviewed by Alexander Kel, Igor Rogozin, and Vladimir A. Kuznetsov.

**Electronic supplementary material:**

The online version of this article (doi:10.1186/s13062-017-0191-4) contains supplementary material, which is available to authorized users.

## Background

Oil palm is in the genus *Elaeis* of family Arecaceae. The genus has two species - *E. guineensis* (African oil palm) and *E. oleifera* (American oil palm). There are three fruit forms of *E. guineensis*, mainly differing in their shell thickness - *dura* (thick shell), *tenera* (thin shell) and *pisifera* (no shell). The African oil palm is by far the most productive oil crop [1] in the world, with estimated production in year 2015/2016 of 61.68 million tonnes, of which the Malaysian share was 19.50 million tonnes [2]. Palm oil constitutes ~34.35% of the world’s production of edible oils. Globally, palm oil is mainly produced from *E. guineensis* in the *tenera* fruit form. *E. oleifera* is not used commercially due to its low yield. However, it is more disease-resistant and can grow in areas where cultivating *guineensis* is not feasible, e.g., Central-Southern America. Even then, it is mainly planted as a backcross to *guineensis* (interspecific hybrid) to increase the yield. Nevertheless, it has economically valuable traits which plant breeders wish to introgress into *guineensis*, such as a more liquid oil with higher carotenoid and vitamin E contents, disease resistance and slow height increment [1].

The importance of oil palm has resulted in interest to sequence its transcriptomes and genome. Initial efforts were based on expressed sequence tags (ESTs) [3], but the technique, while useful for tagging expressed genes, only provided partial coverage of the coding regions and genome. Next, GeneThresher™ technology was applied to selectively sequence hypomethylated regions of the genome [4]. The oil palm *AVROS pisifera* genome sequence was subsequently released in 2013 [5], and this facilitated completion of the draft oil palm *dura* genome [6]. With the genome sequence [5], coupled with genetic and homozygosity mapping via sequencing, the *SHELL* gene was identified [7]. This facilitated an efficient genetic test to distinguish between the *dura, pisifera* and *tenera* fruit forms. Subsequently, the *VIRESCENS* gene, which regulates the fruit exocarp color [8], and the *MANTLED* gene, which causes tissue culture abnormality [9], were also discovered. Accurate genome annotation was critical for the identification of these genes, and will be crucial for increasing oil palm productivity.

First gene prediction pipelines appeared in the 1990s. In 1997, mathematicians from Stanford developed the Genscan [10] software, followed by a steady stream of specially designed tools to navigate the complexity of various genomes. Combining multiple predictors led to the development of automated pipelines integrating various types of experimental evidence [11]. A major limitation shared by many approaches is their relatively poor performance in organisms with atypical distribution of nucleotides [12–15]. The GC_3_ content of the genes plays an important role, as GC_3_-rich genes in grasses can be better predicted by transcriptome-based rather than homology-based methods [16]. Accurate gene prediction is one of the most important challenges in computational biology, as the prediction quality affects all aspects of genomics analysis.

In our effort to overcome the lack of precision in many predictive models, we developed a computational framework to generate high quality gene annotations for oil palm. The framework uses a combination of the Seqping [17] pipeline developed at the Malaysian Palm Oil Board (MPOB), and the Fgenesh++ [18] pipeline by Softberry. Individual components of the framework were trained on known genes of plants closely related to the oil palm, such as the date palm, to identify the most suitable parameters for gene prediction. The best gene model for each locus was selected to establish a representative “high confidence” gene set. Genes associated with important agronomical traits, namely 42 fatty acid biosynthetic genes and 210 candidate resistance genes, were also identified. The gene information and annotations, made available in an oil palm annotation database, will be an important resource for breeding disease and stress resistant palms with enhanced productivity. This paper describes the identification and characterization of a “high confidence” set of 26,059 oil palm genes that have transcriptome and RefSeq support, and bioinformatics analysis of the genes, including comparative genomics analysis, and database and tool development.

## Methods

### Datasets

We used the *E. guineensis* P5-build of an *AVROS pisifera* palm from Singh et al. [5], which contained 40,360 genomic scaffolds (N50 length: 1,045,414 nt; longest length: 22,100,610 nt; and shortest length: 1992 nt). The *E. guineensis* mRNA dataset is a compilation of published transcriptomic sequences from Bourgis et al. [19], Tranbarger et al. [20], Shearman et al. [21, 22], and Singh et al. [7], as well as 24 tissue-specific RNA sequencing assemblies from MPOB submitted to GenBank in BioProject PRJNA201497 and PRJNA345530 (see Additional file 1), and oil palm expressed sequence tags downloaded from the nucleotide database in GenBank. This dataset was used as transcriptome evidence, and to train the Hidden Markov Model (HMM) for gene prediction.

### Fgenesh++ gene prediction

Fgenesh++ (Find genes using Hidden Markov Models) [18, 23] is an automatic gene prediction pipeline, based on Fgenesh, a HMM-based ab initio gene prediction program [24]. We used oil palm genomic scaffolds to predict the initial gene set, applying the Fgenesh gene finder with generic parameters for monocots. From this set, we selected a subset of predicted genes that encode highly homologous proteins (using BLAST with E-value <1.0E-10) to known plant proteins from the NCBI non-redundant (NR) database. We computed the optimized gene-finding parameters using this subset of predicted oil palm genes as the training set, and executed the Fgenesh++ pipeline to annotate the genes in the genomic scaffolds. The Fgenesh++ pipeline considered all available supporting data, such as the *E. guineensis* mRNA dataset and homologous protein sequences. NR plant, and specifically, palm transcripts were mapped to the oil palm genomic scaffolds, identifying a set of potential splice sites. Plant proteins were also mapped to the oil palm genomic scaffolds and high scoring matches were selected to generate protein-supported gene predictions. This ensured that only highly homologous proteins were used in gene identification.

Amino acid sequences from the predicted oil palm genes were then compared to the protein sequences from plant NR database using the ‘bl2seq’ routine, with the similarity considered significant if it had blast percent identity ≥50, blast score ≥ 100, coverage of predicted protein ≥80% and coverage of homologous protein ≥80%. BLAST analysis of the predicted sequences was also carried out against the *E. guineensis* mRNA dataset, using an identity cutoff of >90%. Predictions that have both NR plant RefSeq and *E. guineensis* mRNA support were selected for further analysis.

### Seqping gene prediction

Seqping [17], a customized gene prediction pipeline based on MAKER2 [25], was developed by MPOB. Full-length open reading frames (ORFs) were identified from the *E. guineensis* mRNA dataset described above, using the EMBOSS *getorf* program. ORFs between 500 and 5000 nt were selected to minimize potential prediction errors. Using BLASTX [26] search, selected ORFs with E-values <1E-10 were considered significantly similar to the RefSeq plant protein sequences. ORFs with BLASTX support were clustered using BLASTClust and CD-HIT-EST [27], and subsequently filtered using the TIGR plant repeat database [28], GIRI Repbase [29], and Gypsy Database [30] to remove ORFs similar to retroelements. The resulting set of ORFs was used as the training set to develop HMMs for three modellers, GlimmerHMM [31, 32], AUGUSTUS [33] and SNAP [34] programs, which were subsequently used for gene predictions. Seqping uses MAKER2 [25] to combine predictions from the three modelers. All programs used the default parameters in Seqping. The predicted sequences were compared to the RefSeq [35] protein sequences and *E. guineensis* mRNA dataset by BLAST. Predictions that have NR plant RefSeq and *E. guineensis* mRNA support (E-value cutoff: 1E-10) were selected for further analysis.

### Integration of Fgenesh++ and Seqping gene predictions

To increase the accuracy of annotation, predictions independently made by the Seqping and Fgenesh++ pipelines were combined into a unified prediction set. All predicted amino acid sequences were compared to protein sequences in the NR database using BLAST (E-value cutoff: 1E-10). ORF predictions with <300 nucleotides were excluded. Predicted genes from both pipelines in the same strand were considered overlapping if the shared length was above the threshold fraction of the shorter gene length. A co-located group of genes on the same strand was considered to belong to the same locus if every gene in the group overlapped at least one other member of the same group (single linkage approach) at the selected overlap threshold. Different overlap thresholds, from 60% to 95% in 5% increments, were tested to determine the best threshold value, simultaneously maximizing the annotation accuracy and minimizing the number of single-isoform loci. Protein domains were predicted using PFAM-A [36, 37] (release 27.0) and PfamScan ver. 1.5. The coding sequences (CDSs) were also compared to NR plant sequences from RefSeq (release 67), using the *phmmer* function from the HMMER-3.0 package [38, 39]. To find the representative gene model and determine its function for each locus, we selected the lowest E-value gene model in each locus and the function of its RefSeq match. We excluded hits with E-values >1E-10, as well as proteins that contained words “predicted”, “putative”, “hypothetical”, “unnamed”, or “uncharacterized” in their descriptions, keeping only high-quality loci and their corresponding isoforms. Loci without the RefSeq match were discarded. The CDS in each locus with the best match to the RefSeq database of all plant species was selected as the *best representative* CDS for the locus. Gene Ontology (GO) annotations were assigned to the palm genes, using the best NCBI BLASTP hit to *Oryza sativa* sequences from the MSU rice database [40] at an E-value cutoff of 1E-10.

### Intronless genes

Intronless genes (IG) were identified as mono-exonic genes containing full-length ORFs, as specified by the gene prediction pipeline. The same approach was applied to five other genomes: *A. thaliana* (TAIR10) [41], *O. sativa* (MSU 6.0) [40], *S. bicolor* (Phytozome 6.0), *Z. mays* (Phytozome) and *Volvox carteri* (Phytozome 8.0) [42]. Lists of non-redundant IG from all six genomes were obtained, and the oil palm IG were compared to them using BLASTP (E-value cutoff: 1E-5). The protein sequences of the IG were also mapped to all NCBI genes in the archaea, bacteria and eukaryote kingdoms using BLASTP with the same cutoff.

### Resistance (R) genes

All curated plant resistance (R) genes were downloaded from the database PRGdb 2.0 [43]. A local similarity search of known plant resistance genes and oil palm gene models was done using the BLASTP program with E-value ≤1E-5. TMHMM2.0 [44] was used to find predicted transmembrane helices in the known R genes, as well as in the oil palm candidate R genes, and these results were used to classify the R genes. Domain structures of the known and oil palm candidate R genes were identified using InterProScan. All the domains found were used to classify the candidate R genes according to the PRGdb classification. To be considered an R gene, the gene had to contain all the domains found in known R genes of its class. Our selection was validated on the published “resistance” gene motifs [45–49] and each class further validated via multiple sequence alignment and phylogenetic tree, using the ClustalW [50] and MEGA6 [51] programs, respectively. The same procedure was used to identify R genes in *A. thaliana* [41], *O. sativa* [40], *S. bicolor*, *Z. mays* and *V. carteri* genomes. Distribution of coiled-coil (CC) – nucleotide binding site (NBS) – leucine-rich repeat (LRR) or CNL class R genes across 16 chromosomes of the EG5 genome build [5] was conducted to identify physical clustering. A cluster of R genes is defined as two CNL genes located less than 200 kb apart, with no more than eight non NBS-LRR genes in between them [52, 53].

### Fatty acid (FA) biosynthesis genes

*A. thaliana, O. sativa, Z. mays, Glycine max* and *Ricinus communis* amino acid sequences corresponding to 11 FA biosynthesis genes were obtained from KEGG [54]. The corresponding amino acid sequences for another three genes, oleoyl-phosphatidylcholine desaturase [FAD2], linoleoyl-phosphatidylcholine desaturase [FAD3], acyl-acyl carrier protein (ACP) thioesterase [FATB], were obtained from journals [55–58]. These sequences were compared to oil palm gene models using Exonerate [59] with the “protein2dna” alignment model parameter. The oil palm gene models were annotated using BLASTX against the RefSeq database. Conserved domains of these genes were identified using InterProScan [60] against the HMMPfam database [36, 61]. Corresponding protein sequences of candidate oil palm FA biosynthesis genes and FA biosynthesis genes from other organisms were aligned using the ClustalW program. The catalytic residues and conserved motifs of the amino acid sequences of the corresponding candidate FA biosynthesis genes were identified from literature [62–73]. Sequences of identified FA genes having more than one copy were extracted with additional flanking regions of 10 Mb upstream and downstream to check for genome duplication using the PROmer [74] software with default parameters.

### Expression analysis

To estimate the expression of FA biosynthesis genes, two Illumina HiSeq 2000 libraries each of mesocarp and kernel samples in NCBI BioProject PRJNA245226 [5], were read-mapped to the P5-build of the oil palm genome using the Tuxedo suite [75, 76]. Fragments Per Kilobase of transcript per Million mapped fragments (FPKM) was calculated, with the expression of each gene the mean of measures from two biological replicates. Expressions of genes in root, leaf, leaf apex and flower from BioProject PRJNA201497 were determined by mapping two Roche 454 sequencing transcriptome data for each tissue using the same method.

### Comparative genomics

To identify the orthologs of FA biosynthesis and R genes in oil palm sequences, OrthoMCL2.0 [77] was used with its default parameters to construct orthologous groups across three sets of gene models: *E. guineensis*, *A. thaliana* and *Z. mays*. The corresponding protein sequences of these genes were confirmed with BLASTP [26] searches against the NCBI NR database with default parameters. Protein members of the cluster sequences were aligned by two methods, Muscle [78] and MAFFT [79] version 7. Protein domain sequences were identified using Pfam [37], InterPro [80], ScanProsite [81] and NCBI CDD [82]. To get an overview of the relationships between selected orthologous genes, phylogenetic trees were constructed using MEGA6 [51] and MAFFT [83]. All programs were used with their default settings.

## Results and discussion

### Gene models

A variety of tools has been developed for prediction and annotation of protein-coding genes, such as Fgenesh++ [18], MAKER-P [84], Gramene [85], GeneMark [86, 87], and Ensembl [88]. Plant genomes (such as *A. thaliana, Medicago truncatula, O. sativa, E. guineensis, Fragaria vesca* and others) are generally annotated using a combination of evidence-based gene models and ab initio predictions [6, 89–92]. The first version of the oil palm genome [5], which is from the *AVROS pisifera* palm, was published in 2013 with assembled sequences representing ~83% of the 1.8 Gb-long genome. Using this assembly, we predicted gene models by combining output from the two pipelines, Fgenesh++ and Seqping [17].

**Fig. 1:**
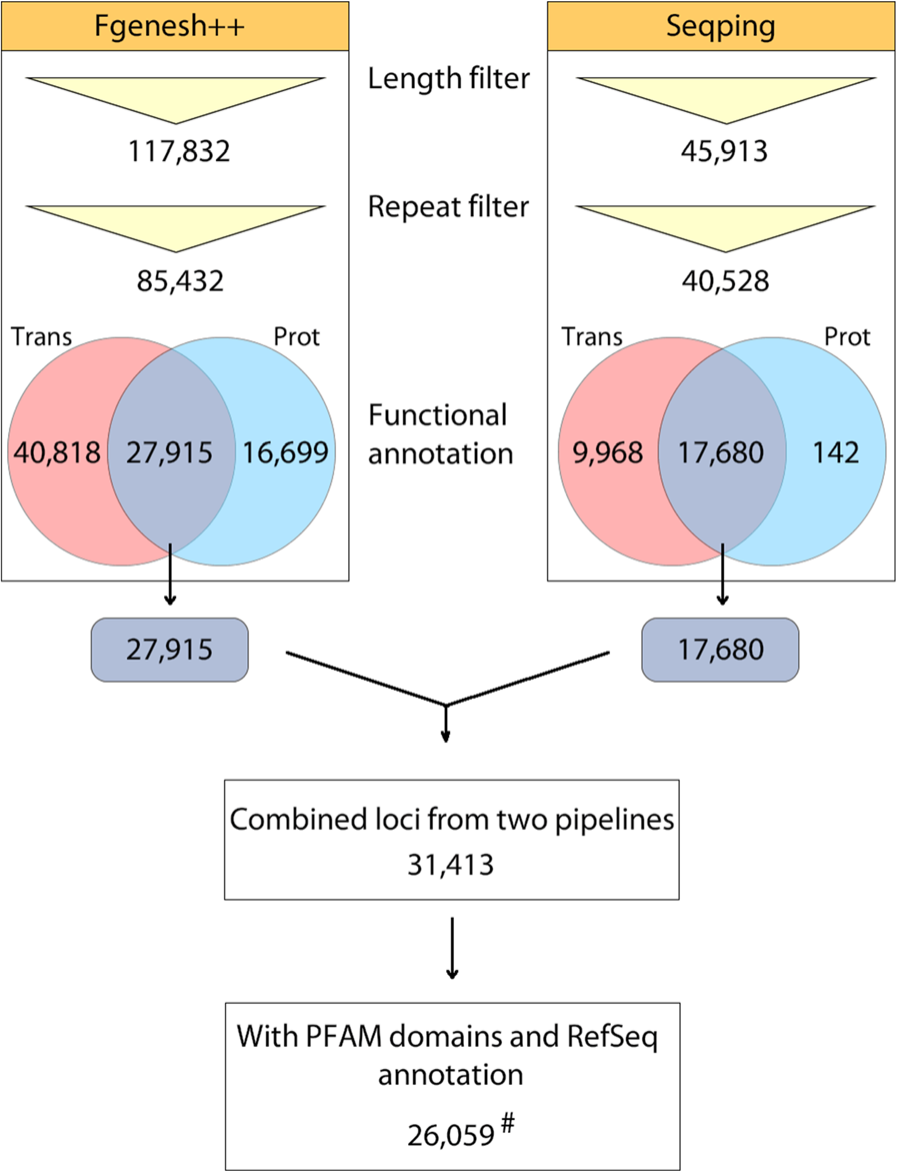
Integration workflow of Fgenesh++ and Seqping gene predictions. Trans – Gene models with oil palm transcriptome evidence; Prot – Gene models with RefSeq protein evidence. # The 26,059 gene models formed the representative gene set that was used for further analysis. The representative gene set was also used to identify and characterize oil palm IGs, R and FA biosynthesis genes

Previous studies of five ab initio pipelines, Fgenesh++, GeneMark.hmm, GENSCAN, GlimmerR and Grail, to evaluate gene prediction precision showed that Fgenesh++ produced the most accurate maize genome annotations [23]. Fgenesh++ is a common tool for eukaryotic genome annotation, due to its superior ability to predict gene structure [93–96]. In the oil palm genome, Fgenesh++ predicted 117,832 whole and partial-length gene models of at least 500 nt long. A total 27,915 Fgenesh++ gene models had significant similarities to the *E. guineensis* mRNA dataset and RefSeq proteins (Fig. 1).

To improve the coverage and accuracy of gene prediction, and to minimize prediction bias, Seqping, which is based on the MAKER2 pipeline [25], was also used. Seqping is an automated pipeline that generates species-specific HMMs for predicting genes in a newly sequenced organism. It was previously validated using the *A. thaliana* and *O. sativa* genomes [17], where the pipeline was able to predict at least 95% of the Benchmarking Universal Single-Copy Orthologs’s (BUSCO) [97] plantae dataset (BUSCO provides quantitative measures for the assessment of gene prediction sets based on evolutionarily-informed expectations of gene content from near-universal single-copy orthologs [97]). Seqping demonstrated the highest accuracy compared to three HMM-based programs (MAKER2, GlimmerHMM, and AUGUSTUS) with the default or available HMMs [17]. The pipeline was used to train the oil palm specific HMMs. This was done by identifying 7747 putative full-length CDS from the transcriptome data. Using this set, the oil palm-specific HMMs for GlimmerHMM [31, 32], AUGUSTUS [33], and SNAP [34] were trained. These HMMs were used in MAKER2 to predict oil palm genes. The initial prediction identified 45,913 gene models that were repeat-filtered. A total 17,680 Seqping gene models had significant similarities to the *E. guineensis* mRNA dataset and RefSeq proteins (Fig. 1).

**Fig. 2:**
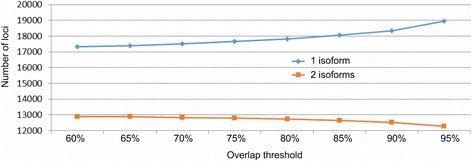
Overlap thresholds using the rate of increase of single-isoform loci. Based on the widening divergence at 85%, the level was selected as the overlap threshold

The 27,915 and 17,680 gene models from Fgenesh++ and Seqping respectively were then combined. Since the ratio of single-gene model to multi-gene model loci increased more rapidly above the 85% overlap between two loci (Fig. 2 and Additional file 2: Table S1), we set this value as the overlap threshold. Gene models that had an overlap ≥85% were grouped into a locus. This threshold allowed us to minimize false positives in merging loci, while maximizing true positives in joining gene models into one locus. The gene models in a single locus must also be predicted from the same strand. Examples of these overlaps are shown in Additional file 3: Figures S1a and S1b. 31,413 combined loci (Additional file 2: Table S1) in 2915 scaffolds were obtained, of which 26,087 contained gene models with PFAM domains and RefSeq annotations. Of them, 13,228 contained one ORF, 12,111 two, and 748 three or more. For every locus, the CDS with the best match to plant proteins from the RefSeq database was selected as its *best representative* CDS.

The genomic scaffolds containing predicted genes were screened by MegaBLAST search against the RefSeq Representative Genome Database (E-value cutoff: 0; hits to *E. guineensis* excluded). If the best BLAST hits were represented by bacterial or plastid plant genomes, the scaffolds were marked as potential contaminants. Forty three potential contaminant scaffolds were identified and checked manually. The scaffolds were also compared to the *oleifera* genome, RNA-seq data and the latest Pisifera genome builds that MPOB uses internally. Scaffolds with no support in all three levels were removed from the final dataset, 24 scaffolds containing 28 loci. The remaining representative CDS for 26,059 genomic loci (the “high quality” dataset) are supported by the oil palm transcriptome and RefSeq data. The sequences and annotations of the 26,059 genes are available in the PalmXplore system (http://palmxplore.mpob.gov.my). PalmXplore is an integrated database system that allows researchers to search, retrieve and browse oil palm gene information and associated functional annotations using a series of search engines. The system is also linked to Blast tools and the oil palm palm genome browser (MYPalmViewer; http://gbrowse.mpob.gov.my/). Screenshots of the system are available in Additional file 4.

**Fig. 3:**
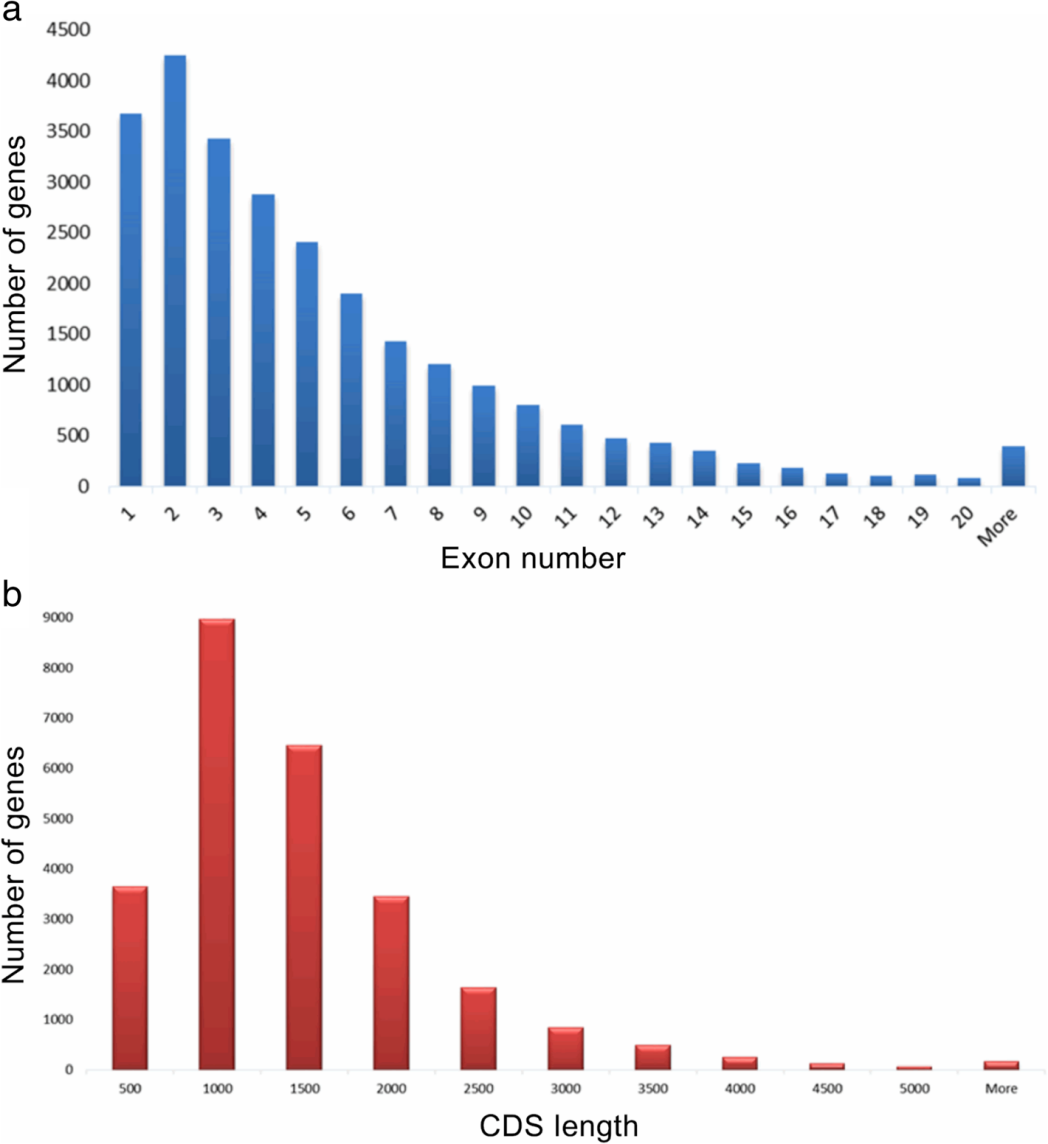
Distribution of oil palm gene models. **a** Number of genes vs. number of exons per gene **b** Number of genes vs lengths of CDS

Gene structure analysis of the high quality dataset showed that 14% were intronless and 16% contained only two exons. 395 genes had more than 20 exons. Further analyses on these genes using BLASTX (E-value cutoff: 1E-5) to determine their identity and exon numbers, showed that 366 had alignment coverage above 90% with the RefSeq [35] genes. The number increased to 384 genes when the cutoff was reduced to at least 80% coverage. The two oil palm genes with the largest exon number (57 exons) were p5.00_sc00063_p0008 and p5.00_sc00076_p0105. Detailed examination of gene p5.00_sc00063_p0008 showed it is similar to serine/threonine-protein kinase TOR from *Musa acuminate*, *Vitis vinifera*, *Citrus sinensis* and *Theobroma cacao,* which also have 57 exons. Interestingly, the oil palm translation activator GCN1 (p5.00_sc00076_p0105) was similar to the genes in *Phoenix dactylifera*, *V. vinifera*, *O. sativa* and *M. acuminate* with 60 exons. The distributions of exons per gene and CDS lengths are shown in Fig. 3a and b respectively. Evolutionary conservation of gene structure was previously described for several species and gene families [98, 99]. For example, it was estimated that in mouse and human, 86% of the orthologous gene pairs have the same number of coding exons [100].

BUSCO analysis [97] of the high quality dataset showed 90.44% of the 429 eukaryotic BUSCO profiles available. By comparing to 1440 *embryophyta* BUSCO profiles (Additional file 2: Table S2), 85.76% of the BUSCO genes were found in the predicted gene models, including 81.25% as complete BUSCO genes, thus quantifying the completeness of the oil palm genome annotation. By comparison, the first set of gene prediction by Singh et al. [5] in 2013 had matches to only 60.35% of the *embryophyta* BUSCO profiles, with 41.60% as complete BUSCO genes, indicating a big improvement in the latest gene models. Also, for each gene in the current and 2013 annotation, we compared the best match to the plant RefSeq database using the NCBI BLASTP program. The new *pisifera* annotation has higher identity to the RefSeq proteins than the old one. The high quality dataset also had better predictions than the 36,105 gene models identified in the *dura* genome [6]. BUSCO analysis (Additional file 2: Table S2) shows that the *pisifera* annotations contain 53% more complete (1170 vs. 765), 55% less fragmented (65 vs. 145), and 61% less missing (205 vs. 530) BUSCO profiles than those from *dura*. The average number of exons in *dura* is 4.3, and in *pisifera* 5.4. The predicted mean CDS length of *dura* (900 nt) is also shorter than *pisifera* (1232 nt).

### Nucleotide composition of oil palm genes

One important characteristic of a genome is the frequency of guanine and cytosine occurring in the third codon position, GC_3_, which is defined as $$ \frac{C_3+{G}_3}{\left(\raisebox{1ex}{$L$}\!\left/ \!\raisebox{-1ex}{$3$}\right.\right)} $$, where *L* is the length of the coding region, *C*_3_ the number of cytosines, and *G*_3_ the number of guanines in the third position of codons in the coding region [16]. Two types of GC_3_ distribution have been described - unimodal and bimodal [16, 101, 102]. Genes with high and low GC_3_ peaks have distinct functional properties [102]. GC_3_-rich genes provide more targets for methylation, exhibit more variable expression, more frequently possess upstream TATA boxes and are predominant in stress responsive genes. Different gene prediction programs have variable bias to different classes of genes, but GC_3_-rich genes are reported to be especially hard to predict accurately [103]. The distribution of GC_3_ is bimodal in grasses and warm-blooded vertebrates, and unimodal in other species sequenced to date [104].

**Fig. 4:**
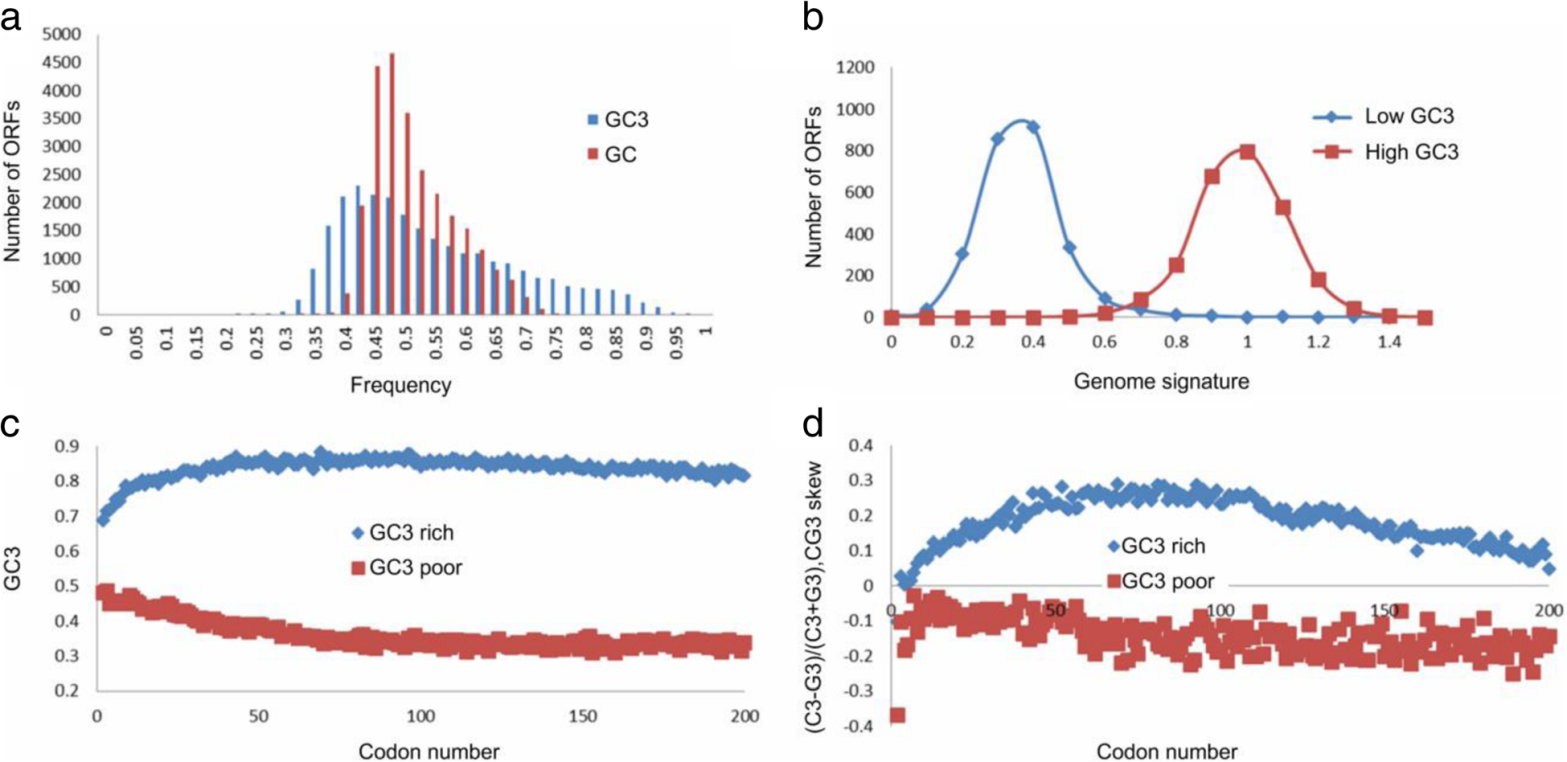
GC_3_ distribution in oil palm gene models. **a** GC (*red*) and GC_3_ (*blue*) composition of coding regions of *E. guineensis*. **b** Genome signature for GC_3_-rich and -poor genes. **c** GC_3_ gradient along the open reading frames of GC_3_-rich and -poor genes. **d** CG_3_ skew gradient along the open reading frames of GC_3_-rich and -poor genes. Figures **c** and **d**: x-axis is number of codons in coding sequence. Figure **d**: C_3_ and G_3_ is frequency of cytosine or guanine in third position of codon. CG_3_ is frequency of cytosine and guanine in third position of codon

The distribution of GC_3_ in oil palm is unimodal with a long tail towards high values of GC_3_. Figure 4a shows the distribution of GC_3_ in the high quality dataset. We ranked all genes by their GC_3_ contents and designated the top 10% (2606 ORFs) as GC_3_-rich (GC_3_ ≥ 0.75286) and the bottom 10% as GC_3_-poor (GC_3_ ≤ 0.373239). Two of the remarkable features that distinguish GC_3_-rich and -poor genes are the gradients of GC_3_ and CG_3_-skew, defined as ${CG}_3^{skew}=\frac{C_3-{G}_3}{C_3+{G}_3}$, where C_3_ and G_3_ are the frequencies of cytosines or guanines in the third position of the codon, correspondingly. An increase in the ${CG}_3^{skew}$ from 5' to 3' has been linked to transcriptional efficiency and methylation status [16, 102, 105] of the GC_3_-rich genes. Figure 4c and d show the positional gradients of nucleotide composition. The GC_3_ content of GC_3_-rich genes increases from the 5' to 3' end of the gene, but decreases in GC_3_-poor genes. Despite the relatively small number of GC_3_-rich genes in the oil palm genome, there are characteristic patterns of positional gradients (Fig. 4c and d) near the predicted start of translation, as also found in other well-annotated genomes [16].

The dinucleotide CG relative abundance (a.k.a. “genomic signature”) is defined as ${\rho}_{CG}=\frac{f_{CG}}{f_C{f}_G},$ where *f*_*x*_ is the frequency of a (di)nucleotide *x* [106]. Similar to grasses, and other previously analyzed plant and animal species [16, 102], the oil palm genome signature differs for GC_3_-rich and GC_3_-poor genes (Fig. 4b). The GC_3_-rich genes are enriched and the GC_3_-poor genes depleted in the number of CpG sites that are potential targets for methylation. Gene ontology analysis shows that many of the GC_3_-rich genes are stress-related, while many of the GC_3_-poor genes have housekeeping functions (see GO annotation in Additional file 2: Table S3). The depletion of CpGs in GC_3_-poor genes is consistent with their broad constitutive expression [16]. This analysis is based on the classification described above where the GC_3_-rich genes were defined as the top 10% genes with the highest GC_3_ content, and the GC_3_-poor genes the bottom 10% of all genes with the lowest GC_3_ content. If there is no relationship between nucleotide composition and GO categories, the distribution of genes in the GO categories would be the same for all the genes in the entire genome. However, the goodness-of-fit test shows that, for example, in the GO categories ‘response to abiotic stimulus’, ‘response to endogenous stimulus’ and ‘secondary metabolic process’, the number of genes in GC_3_-rich and -poor categories differ from uniform distribution at *p*-value = 6.12E-13, 6.68E-08 and 1.56E-06 respectively.

We calculated the distribution of nucleotides in the oil palm coding regions. The following models of ORF were considered: *Multinomial* (all nucleotides independent, and their positions in the codon not important), *Multinomial* position-specific and *First order three periodic Markov Chain* (nucleotides depend on those preceding them in the sequence, and their position in the codon considered). Additional file 2: Tables S4-S7 show the probabilities of nucleotides A, C, G and T in GC_3_-rich and -poor gene classes. Note that both methods predict GC_3_-poor genes with greater imbalance between C and G, than GC_3_-rich genes (0.05 vs. -0.1). This is consistent with the prior observation [102] that GC_3_-rich genes have more targets for methylation than GC_3_-poor genes, and that some cytosine nucleotides can be lost due to cytosine deamination.

GC_3_-rich and -poor genes differ in their predicted lengths and open reading frames (Additional file 2: Table S8). The GC_3_-rich genes have gene sequences and ORFs approximately seven times and two times shorter, respectively, than the GC_3_-poor genes. This is consistent with the findings from other species [16, 101, 102]. It is important to note that GC_3_-rich genes in plants tend to be intronless [16].

### Intronless genes (IG)

**Fig. 5:**
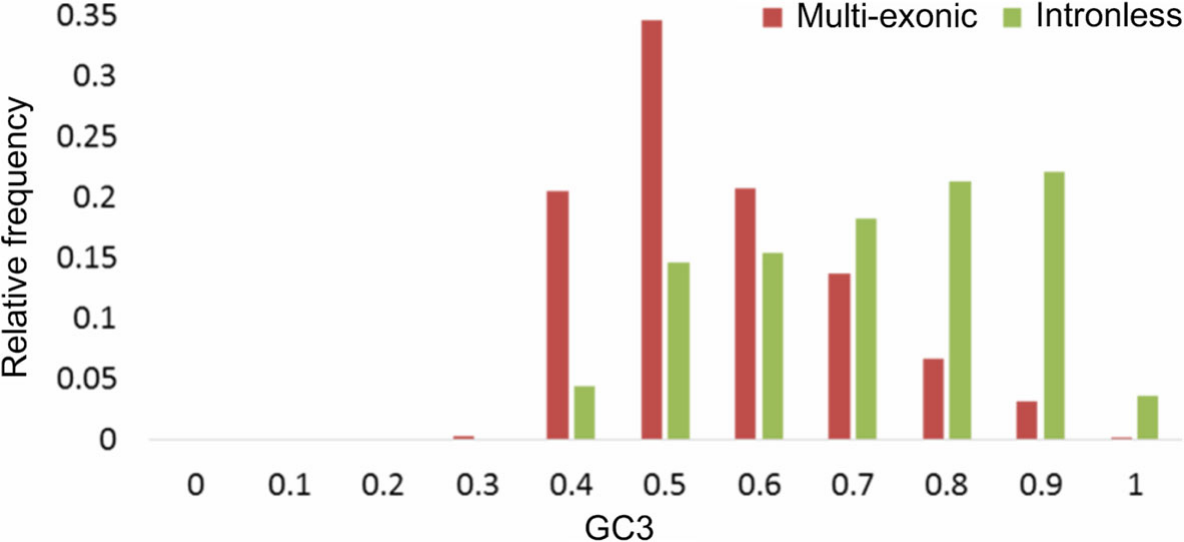
GC_3_ contents of oil palm intronless and multi-exonic genes

Intronless genes (IG) are common in single-celled eukaryotes, but only a small percentage of all genes in metazoans [107, 108]. Across multi-cellular eukaryotes, IG are frequently tissue- or stress-specific, GC_3_-rich with their promoters having a canonical TATA-box [16, 102, 107]. Among the 26,059 representative gene models with RefSeq and oil palm transcriptome evidence, 3658 (14.1%) were IG. The mean GC_3_ content of IG is 0.668 ± 0.005 (Fig. 5), while the intron-containing (a.k.a. multi-exonic) genes’ mean GC_3_ content is 0.511 ± 0.002, in line with the estimates for other species. IG are over-represented among the GC_3_-rich genes (GC_3_ > =0.75286). 36% of intronless genes are GC_3_-rich, in comparison with an overall 10% in all oil palm genes (Chi-squared test *p*-value < 10^−16^). Intronless genes constitute 51% of the GC_3_-rich genes. Their CDS are, on average, shorter than multi-exonic CDS: 924 ± 19 nt vs. 1289 ± 12 nt. On average, there is one intronless gene per 9.5 multi-exonic genes on any scaffold containing intronless genes. There is no difference in nucleotide composition and CpG frequency between short scaffolds that contain intronless genes, multi-exonic genes and no genes.

The distribution of IG in the whole genome is different for various functional groups [16, 108]. For example, in the oil palm genome, 29% of the cell-signaling genes are intronless, compared to just 1% of all tropism-related genes (Additional file 2: Table S9). The distribution of genes by GO categories is similar to that in *O. sativa*. It has been shown that in humans, mutations in IG are associated with developmental disorders and cancer [108]. Intronless and GC_3_-rich genes are considered to be evolutionarily recent [16] and lineage-specific [107], potentially appearing as a result of retrotransposon activity [108, 109]. It is reported that 8–17% of the genes in most animals are IG, ~10% in mice and humans [107] and 3–5% in teleost fish. Plants have proportionately more IG than animals, 20% in *O. sativa*, 22% in *A. thaliana* [110], 22% in *S. bicolor*, 37% in *Z. mays*, 28% in foxtail millet, 26% in switchgrass and 24% in purple false brome [111]. We have independently calculated the fraction of IG in *O. sativa*, *A. thaliana*, *S. bicolor* and *Z. mays* using the currently published gene models for each species, with results of 26%, 20%, 23% and 37%, respectively (Additional file 2: Table S10). To establish a reference point, we calculated the fraction of IG in the green algae, *V. carteri*, and found 15.8%. High IG in grasses is not surprising, since they have a clearly bimodal distribution of GC_3_ composition in their coding region, with the GC_3_-peak of this distribution dominated by IG [16].

**Fig. 6:**
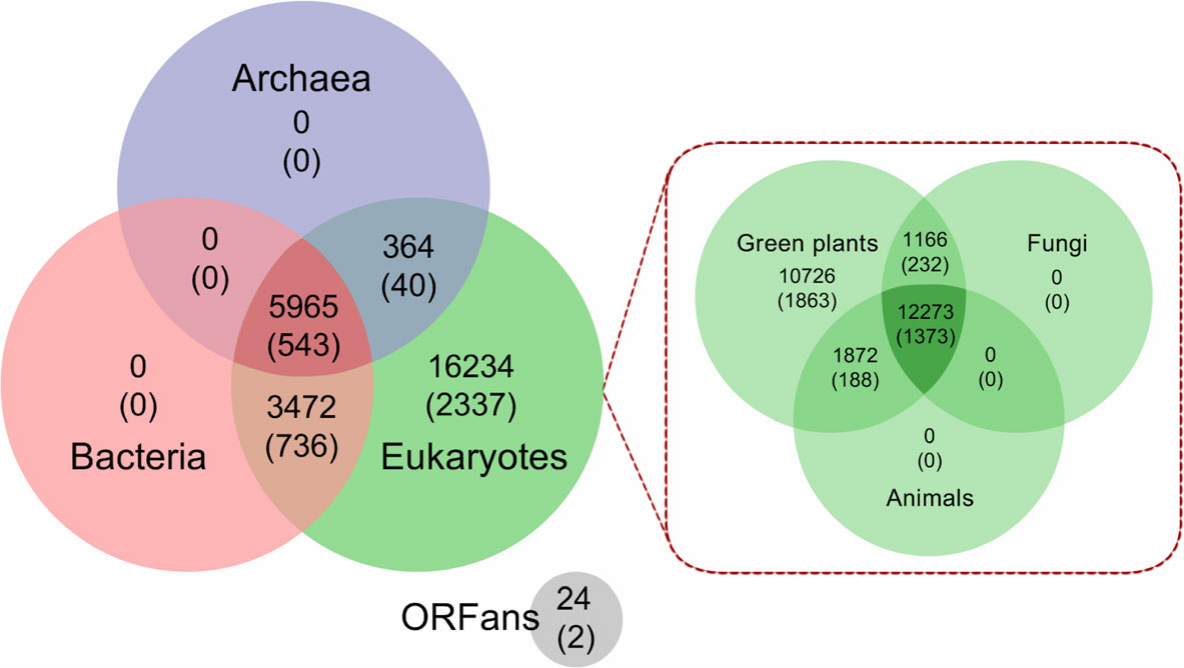
Classification of oil palm intronless genes (IG) in different taxonomy groups. The Venn diagram shows the projections of 26,059 oil palm high quality loci and 3658 oil palm IG (in parenthesis) into three domains of life based on homology, archaea, bacteria and eukaryotes. The sub-diagram shows the distribution of oil palm IG from the eukaryote domain into three major taxonomy groups of life - Green Plants, Fungi and Animals. ORFans refers to the unique sequence that shares no significant similarity with other organisms

Using BLASTP, we found 543 IG (14.84% of oil palm IG) conserved across all the three domains of life: archaea, bacteria and eukaryotes (Fig. 6). These genes are likely essential for survival [112]. A total 736 oil palm IG had homologs only with eukaryotic and bacterial genes, while only 40 IG had homologs with eukaryotic and archaea genes (and not with bacteria). We speculate (due to the extreme growth conditions of archaea [113, 114]) that there are (1) fewer opportunities for horizontal gene transfer from archaea than from bacteria to the oil palm genome, and/or (2) possible ancestral gene loss on the archaeal branch in the process of adaptation. Considering three of the most economically important eukaryotic groups [Metazoa (animals), Fungi and Viridiplantae (green plants)] we observed 1373 oil palm IG shared among them. A significant portion of the oil palm IG (1863) was only homologous to Viridiplantae. These proteins may have evolved, or been regained, only in plants, even as other organisms lost their ancestral genes during evolution [110].

Reciprocal BLAST was carried out to verify the homologies of oil palm candidate IG to produce a set of high confidence oil palm IG. We found 2431 (66.46%) proteins encoded by oil palm IG to have orthologs in *A. thaliana*, *O. sativa* or *Z. mays* that are also intronless, indicating that intronlessness is an ancestral state [115, 116]. In conclusion, from our representative gene models, we estimate that about one-seventh of the genes in oil palm are intronless. We hope that this data will be a resource for further comparative and evolutionary analysis, and aid in understanding IG in plants and other eukaryotic genomes.

### Resistance (R) genes

Plants differ from animals in many aspects, one of them is the lack of an antibody-based immune system. Instead, they have protein-based mechanisms to recognize invading pathogens [117–119]. The genes encoding for such proteins are called “resistance”, or “R” genes. They play an important role in the plant’s early detection and signaling mechanism against biotic and abiotic stresses. Using homology, we identified 210 oil palm candidate R genes from the 26,059 representative gene models with RefSeq and transcriptome evidence (see Additional file 1). This is ~0.80% of the high-quality genes identified in the oil palm genome, a similar ratio to that of an earlier study on the hypomethylated regions of the *E. guineensis* genome, where 52 (0.94%) candidate resistance genes were identified among 5505 gene models [4]. A similar frequency was also observed in *A. thaliana* and *O. sativa* - 0.95% and 0.71% resistance genes, respectively. The oil palm candidate R genes were compared to those in banana (*M. acuminata*) and *O. sativa*, and 693 orthologs (253 in *M. acuminata*, 440 in *O. sativa*) were identified for 204 of the genes.

The candidate genes were divided into six classes by their protein domain structure [43]. Comparison of the distribution of oil palm candidate R genes with such genes identified using the same method in other plants showed that CNL class genes had the highest representation in monocots, with *O. sativa* having the largest percentage (51.8%). *A. thaliana*, which is dicotyledonous, has two additional classes, TNL (Toll/interleukin-1 NBS-LRR) and RPW8-NL, while the colonial green algae *V. carteri* is missing most of the R gene classes in its genome. TNL, the most prevalent class in *A. thaliana*, is predominantly found in dicots [120]. The CNL and TNL classes both belong to the NBS-LRR family [121]. TNL can be differentiated from CNL based on the Toll/interleukin-1 (TIR) receptor domain at the N-terminus structure [120].

**Fig. 7:**
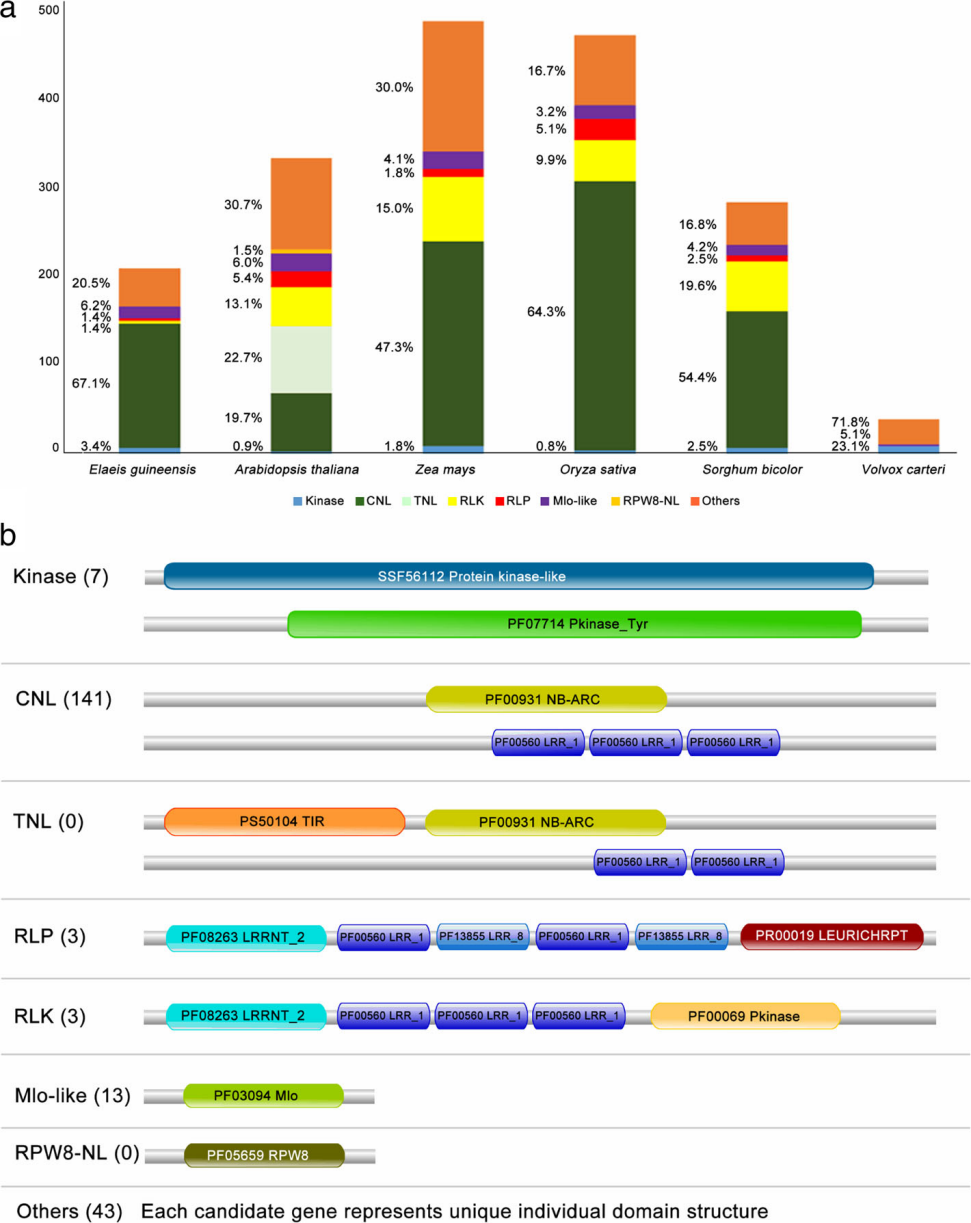
Classification of candidate R genes. **a** Distribution of the genes in oil palm, *A. thaliana*, *Z. mays*, *O. sativa*, *S. bicolor* and *V. carteri* **b** Examples of key domains identified via InterProScan in oil palm candidate R-genes. Number of identified candidate oil palm genes are in brackets

We did not identify any TNL gene in the analyzed monocot genomes, including that of the oil palm. This is in line with Tarr and Alexander [122] who also did not find TNL genes in monocots. It is therefore assumed that R genes in monocots predominantly contain leucine zipper regions that facilitate formation of the conserved CC structure at the N-terminal of NBS-LRR genes, as previously indicated [123]. The CC domain is required for protein-protein interaction [46] while the LRR domain interacts with the avirulence (Avr) gene product from pathogens to activate the plant defense system [124]. Plants producing specific R genes are resistant to pathogens which produce the corresponding Avr gene products. The fraction of R genes across the plant genome suggests the importance of these genes for both monocots and dicots (Fig. 7a).

CNL genes form the most abundant class in the oil palm genome. A total 141 genes were identified, of which 133 have orthologs in other plants. The remaining eight are unique to the oil palm and may be involved in palm-specific interactions with pathogen Avr gene products. Validation using multiple sequence alignments of the oil palm CNL genes and their orthologs showed a conserved kinase-2 motif with the last residue W (Tryptophan) in the NBS domain in most of the oil palm CNL genes. Of the 141 oil palm CNL genes, only nine do not have the final residue, W. The W residue is highly conserved in non-TIR NBS-LRR genes [120]. The percentage of CNL genes (67.14%) agrees with Staskawicz et al. [125] who reported that the majority of disease resistance genes in plants contain the NBS-LRR domain [126].

Another class of R genes critical for plant defense is the Kinase class. This class contains an intracellular serine/threonine protein kinase (STK) domain which plays an important role in many plant processes, including plant-pathogen interaction [46, 48, 127, 128]. *Pto*, an R gene previously identified in the tomato genome to confer resistance against *Pseudomonas syringae* pv. *tomato* strains, is a Kinase [47, 129]. There are several features defining the *Pto* gene in tomato - Pto activation domain [127], autophosphorylation sites [46, 48, 127], P + 1 loop [129] and N-myristoylation motif [128]. Seven candidate genes in the oil palm genome have the required features. Sequence alignment between the candidate genes and *Pto* revealed several highly conserved sites in the Pto activation domain. However, the third autophosphorylation site in the activation domain had a threonine to glycine mutation (Additional file 3: Figure S2), which was reported to reduce the plant hypersensitive response [127].

The remaining R genes identified were RLP, RLK and Mlo-like. The high-quality oil palm dataset contains three RLP and three RLK genes. Both classes contain the transmembrane and LRR domains [46], but only RLK an additional STK domain (Fig. 7b). RLP and RLK genes function as pattern recognition receptors (PRRs) in the transmembrane region, and are activated in the initial detection of a pathogen in the plant [130, 131]. Other plants, such as *A. thaliana* (9.8% RLK and 4.0% RLP) and *O. sativa* (10.5% RLK and 5.4% RLP), have higher percentages of these genes in their genomes. Since none of the oil palm transcripts used in the gene prediction process originated from stress-related tissues, the number of predicted R-genes may be under-estimated. The actual percentage of these two classes may be higher, but only the six identified RLK and RLP genes were expressed in the transcriptomes used. Oil palm also has 13 candidate Mlo-like genes, classified by having the Mlo domain [46]. The first member of this class, *MLO* gene from barley, was expressed in leaf in response to invasion by a fungal pathogen, *Erysiphe graminis* f sp. *Hordei.* MLO (mildew locus O) is an intrinsic protein with six transmembrane regions [132] while the palm MLO-like candidates have six/seven transmembrane regions.

About 70% of the 210 candidate R genes were distributed across the 16 oil palm chromosomes of the EG5 genome build [5] (Additional file 3: Figure S3). One hundred one of the 141 CNL class R genes were found on 14 of the chromosomes, of which 62 formed 23 clusters by chromosomal location. The highest number of clustered CNL class R genes (42%) were on chromosome 2. R genes in other plants (such as thale cress, flax, barley, lettuce, maize, potato, rice, soybean and tomato) also form location clusters [133]. Plant resistance is determined by (direct or indirect) interaction of the plant R genes with pathogens’ Avr genes, and evolves to adapt to the different forms of Avr genes [124, 134]. Co-located R genes recognize different pathogens and are hypothesised to share function and pathogen recognition systems [133].

Since R genes are important for the plant survival and its surveillance system, the R genes-related domains appear to be evolutionarily conserved across all sequenced plant genomes, including that of oil palm. The high-quality dataset was used to find the necessary domains to classify the R genes into six classes. Identification of these candidate genes is useful for marker development and gene expression studies during infection, especially for basal stem rot, one of the most devastating oil palm diseases in South-East Asia. Comparing the oil palm genome with those of other monocots, it was possible to identify R genes for further functional characterization, and reveal homologous sequences in related crops.

### FA biosynthesis genes

**Fig. 8:**
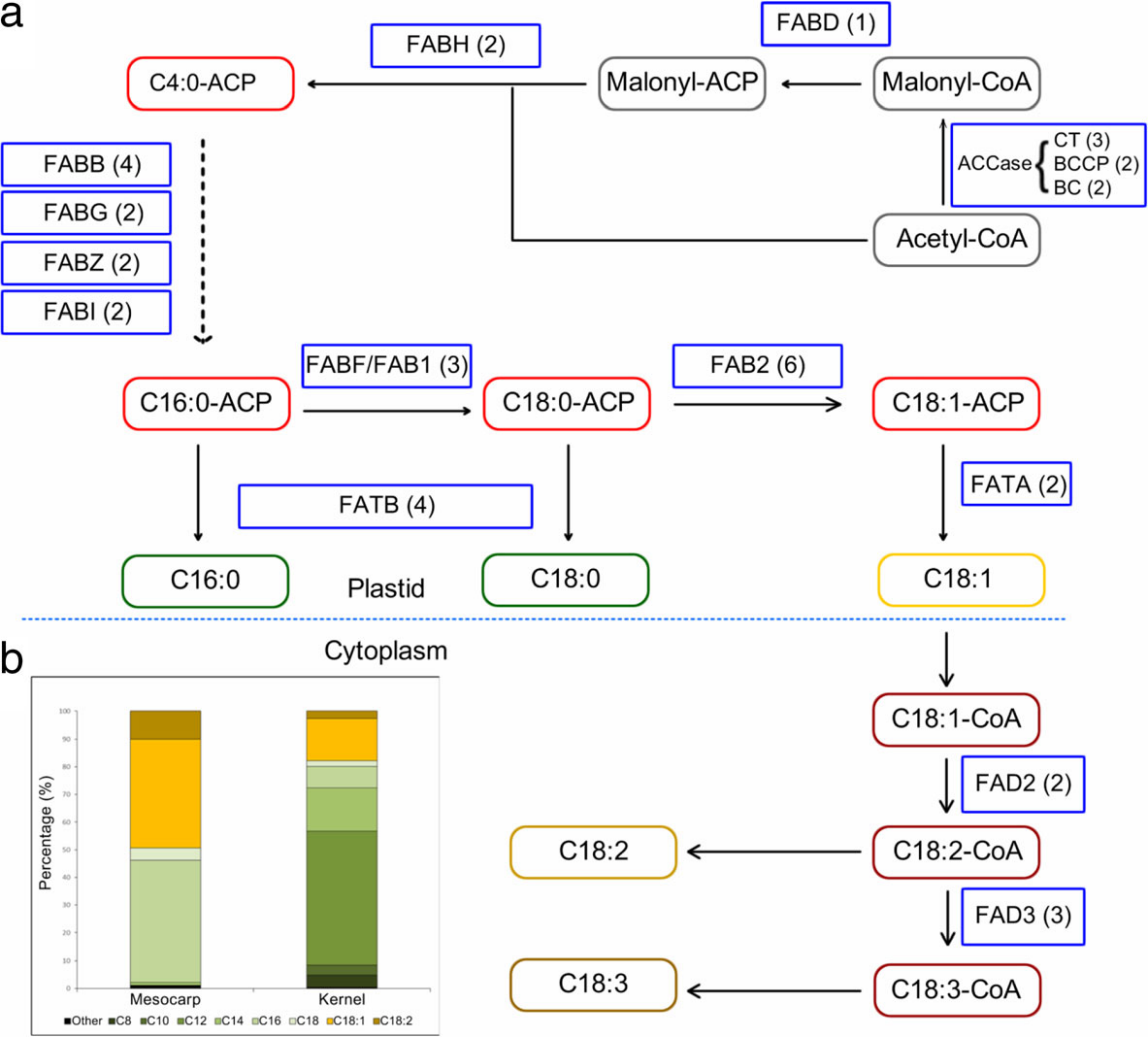
Fatty acid biosynthesis in *E. guineensis* **a** Schematic pathway diagram for fatty acid biosynthesis. Numbers of identified oil palm candidate genes are in brackets. **b** Fatty acid composition in mesocarp and kernel

Oil palm is unique in that it produces different oils with distinct fatty acid profiles in its mesocarp and kernel. The *E. guineensis* mesocarp oil is ~50% saturated (39.2–45.8% palmitic acid [C16:0], 3.7–5.1% stearic acid [C18:0] and 0.9–1.5% myristic acid [C14:0]), 37.4–44.1% monounsaturated (mainly oleic acid [C18:1]) and ~10.5% polyunsaturated (10.2% linoleic acid [C18:2] and 0.3% linolenic acid [C18:3]) [135]. The kernel oil is more saturated, with mainly medium chain fatty acids - lauric ([C12:0], ~48%), myristic (~15%) as well as palmitic (~8%) acid [136]. Kernel oil also contains about 15% oleic acid. The fatty acid compositions also vary noticeably between *E. guineensis* and *E. oleifera* [137, 138]. *E. oleifera* mesocarp oil is typically less saturated (53.5–68.7% oleic acid, 11.9%-26.9% linoleic acid and 0.0%-1.9% linolenic acid) [138]. Forty-two oil palm (*E. guineensis*) genes involved in FA biosynthesis, including two multifunctional acetyl-CoA carboxylases (ACCase), were identified (see Additional file 1). Figure 8a and b show the numbers of oil palm genes in the FA biosynthesis pathway, and oil palm fatty acid composition respectively. The conserved catalytic residues were identified via sequence alignment of the corresponding amino acids (Additional file 3: Figures S4-S15). This method was used by Li et al. [65] to study the candidate FA biosynthesis genes of *Arachis hypogaea* L. Twenty seven FA biosynthesis genes were categorized in 10 classes based on the conserved catalytic residues of their corresponding amino acid sequences, and six identified by their conserved motifs. The remaining nine genes encoding ACCase were mainly classified by homology. Using a 70% identity cut-off, 39 candidate oil palm FA biosynthesis genes had 94 corresponding orthologs in *A. thaliana* (29) and *Z. mays* (65). Overall, these results showed that the classifications were consistent with the annotations of *A. thaliana* and *Z. mays* genes. The three remaining candidate genes, one acyl-ACP thioesterase (*EgFATB_1*) and two stearoyl-ACP desaturases (*EgFAB2_3* and *EgFAB2_4*), were defined as singletons. Closer examination of *EgFAB2_3* indicates that the gene could be truncated, as it had a gap in its genomic region, making it a singleton.

**Fig. 9:**
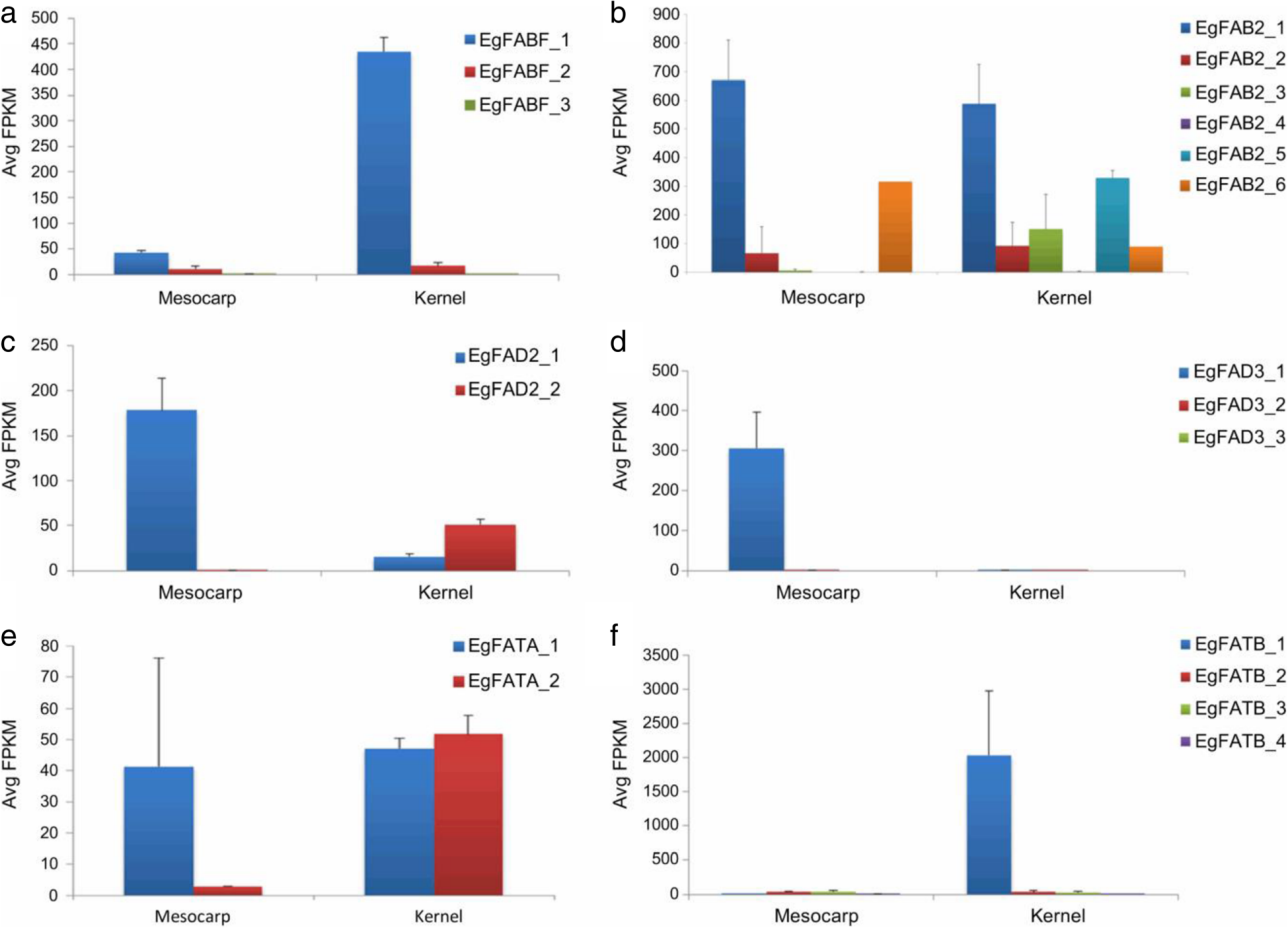
Transcriptome analysis of **a** FABF, **b** FAB2, **c** FAD2, **d** FAD3, **e** FATA and **f** FATB genes in mesocarp and kernel

ACCase plays an important role in de novo FA biosynthesis as it catalyzes the first committed step in the pathway [139]. Analysis of the ACCase genes showed that oil palm contains both the multi-subunit (CT [3 copies], BCCP [2 copies], BC [2 copies]), and multifunctional (2 copies) forms. This agrees with Wan Omar et al. [140]. who reported two distinct forms of ACCase in oil palm. After the first committed step, stepwise addition of two-carbon residues from malonyl-ACP continues until palmitoyl-ACP (C16:0-ACP). C16:0-ACP is then converted to C18:0-ACP by β-ketoacyl-ACP synthase II (FABF) [141]. Biochemical analysis showed that the FABF activity, and level of C18:1 are negatively related with the level of C16:0 [136]. FABF activity in *E. guineensis* was only <50% of several accessions of *E. oleifera* [136]. Although *E. guineensis* has three copies of *FABF*, expression analysis showed a dominant copy in the mesocarp and kernel. *EgFABF_1* is at least 2.8× and 19.2× more highly expressed in mesocarp and kernel respectively than the other two copies (Fig. 9a), suggesting that the conversion of C16:0-ACP to C18:0-ACP is mainly driven by it. Overexpression of this gene copy may drive palm oil to higher oleic acid content. The second copy of *FABF*, *EgFABF_2*, is also expressed in both the mesocarp and kernel samples but at lower levels. This is in line with Umi Salamah et al. [142] who reported that the *FABF* identified, similar to *EgFABF_2* (93% identity at nucleotides level), was also expressed in both mesocarp and kernel samples at relatively higher levels than in other tissues using northern blot analysis. The remaining *EgFABF_3* has very low expression.

Stearoyl-ACP desaturase (encoded by FAB2 [143–145]) plays a crucial role in determining the ratio of saturated to unsaturated C18 fatty acids in plant membranes and storage lipids. Multiple alignment of the corresponding amino acid sequences of the oil palm and other plants *FAB2* genes identified two important motifs (EENRH and DEKRH). In this study, the *FAB2* gene has the highest number of copies among all the FA biosynthesis genes identified. This is not unexpected as oil palm has moderate oleic acid in both its oils, ~40% in its mesocarp oil and ~15% in its kernel oil. FAB2 is a very active enzyme in the developing oil palm mesocarp and any effort to increase the oil oleic acid content may not therefore require upregulating the gene(s) expressing stearoyl-ACP desaturase [135]. Ortholog analysis showed that two oil palm FAB2 genes (*EgFAB2_3* and *EgFAB2_4*) are singletons while four (*EgFAB2_1*, *EgFAB2_2*, *EgFAB2_5* and *EgFAB2_6*) are similar to orthologs in *A. thaliana* and *Z. mays*.

**Fig. 10:**
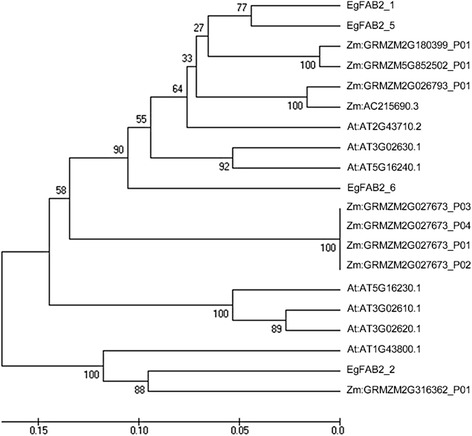
Evolutionary relationship of FAB2 in oil palm (*E. guineensis*), *A. thaliana* and *Z. mays*. Analyses carried out using UPGMA method in MEGA 6 software. Abbreviations: Eg - *E. guineensis*; At - *A. thaliana*; Zm - *Z. mays*

*EgFAB2_1*, *EgFAB2_5* and *EgFAB2_6* are in the same clade as FAB2 genes encoded by AT2G43710 (SSI2), AT5G16240 (S-ACP-DES1) and AT3G02630 (S-ACP-DES5) in *A. thaliana* (Fig. 10). This is interesting because SSI2 is involved in determining the 18:1 pool in *A. thaliana* leaf [146] and has a substrate preference for C18 over C16 fatty acids [146, 147]. Surprisingly, *EgFAB2_1* has the highest expression in the mesocarp and kernel (Fig. 9b), suggesting that it is the dominant copy of the *FAB2* gene, and largely responsible for desaturating C18:0-ACP to C18:1-ACP in de novo FA biosynthesis in the tissues. *EgFAB2_6* also has a relatively high expression in the mesocarp, but is lower in the kernel. The gene may also contribute to the production of C18:1-ACP in the mesocarp, as knocking out *SSI2* in *A. thaliana* only reduced the desaturase activity by 90% [146]. *EgFAB2_3* and *EgFAB2_5* are hardly expressed in the mesocarp, but highly in the kernel, indicating tissue specific expression. Both may play a more important role in C18:1 production in the kernel than mesocarp. *EgFAB2_2* has the highest divergence from the other four genes in the phylogenetic tree, and is orthologous to the *A. thaliana* gene, AT1G43800. Northern analysis of AT1G43800 in *A. thaliana* showed that the gene is not expressed in the leaf, stem, root, flower or silique [146]. This is in line with the oil palm 454-transcriptome data, which showed that *EgFAB2_2* is not expressed in the leaf, root or stalk, with only slight expression in the flower (data not shown). Based on expression analysis, *EgFAB2_2,* like *EgFAB2_3,* and *EgFAB2_5* may play more important roles in C18:1 production in the oil palm kernel than mesocarp. The remaining copy of the *FAB2* gene (*EgFAB2_4*) has very low expression in the mesocarp and kernel.

C18:1 may be further desaturated to polyunsaturated fatty acids in the plastid or endoplasmic reticulum (ER). FAD2 and FAD3, localized in the ER, are responsible for the synthesis of C18:2 and C18:3, respectively, in storage oils. *EgFAD2_1* and *EgFAD3_1* are the dominant copies of *FAD2* and *FAD3*, respectively, that probably drive the desaturation of C18:1 in the mesocarp (Fig. 9c-d). The expression data showed higher FAD2 and FAD3 expression in the mesocarp than kernel, consistent with the fact that the mesocarp oil contains some C18:2 and C18:3, both of which are insignificant in kernel oil.

Acyl-ACP thioesterases terminate de novo chain elongation by hydrolyzing the acyl-groups on acyl-ACP fatty acids [148, 149]. The unesterified fatty acids released are exported to the ER for modification, such as assembly into triacylglycerols and/or membrane lipids. Thioesterases are either FATA or FATB, depending on their specificity for acyl groups - FATA prefers unsaturated and FATB saturated. Six oil palm acyl-ACP thioesterase genes were identified. The corresponding amino acid sequences of the genes contain two conserved motifs, NQHVNN and YRRECG. However, the conserved YRRECG motif in oil palm and other plants differed from the PFAM HMMLogo (Additional file 3: Figures S14 and S15), in line with Voelker et al. [150], who postulated plant thioesterases as a different class of enzymes from those of animals and bacteria. Multiple alignment, BLAST, and ortholog analysis of the corresponding amino acid sequences (Additional file 3: Figure S16) were able to classify *EgFATA_1* and *EgFATA_2* as oleoyl-ACP thioesterase (*FATA*) genes. *EgFATA_1* and *EgFATA_2* are highly similar to experimentally derived oleoyl-ACP thioesterase AAD28187.1 in *E. guineensis* [151], with 97% and 89% BLASTP identity respectively, and to NP_001292940.1 from *J. curcas* (69% identity, 76% positives) and XP_007049712.1 from *T. cacao* (72% identity, 90% positives). Both these sequences have high homology and formed a clade with other characterized plant FATA genes. The remaining four could not be differentiated via sequence analysis but expression data suggested that they function as FATB to hydrolyze saturated acyl-ACPs. *EgFATB_1* is not expressed in the mesocarp but has very high expression in the kernel, indicating that it is mainly involved in fatty acid chain termination in the kernel (Fig. 9f).

As oil palm accumulates 48% C12:0 and 15% C14:0 in its kernel oil, *EgFATB_1* probably encodes for a thioesterase with substrate specificity for medium chains, i.e. lauryl- or myristoyl-ACP thioesterase. *EgFATB_2* and *EgFATB_3*, only moderately expressed in the mesocarp and kernel, are probably involved in the formation of C16:0 since the acid accumulates to ~44% in the mesocarp oil and 15% in the kernel oil. The remaining acyl-ACP thioesterase (*EgFATB_4*) was only detected at very low levels in both the mesocarp and kernel, and may code for stearoyl-ACP thioesterase as palm oil and palm kernel oil only contain 3.7–5.1% [135] and 0.5–5% [152] stearic acid, respectively.

Comparison of the genomic locations of the FA biosynthesis genes in the oil palm genome showed that three genes, namely *EgFABF*, *EgFABH* and *EgFAD3*, showed duplication events (Additional file 3: Figure S17). This is in accordance with the segmental duplications of chromosome arms reported by Singh et al. [5]. The study identified and characterized 42 key genes involved in FA biosynthesis in *E. guineensis*. This is the first study to identify key FA biosynthesis genes in both the oil palm mesocarp and kernel through sequence and gene expression analysis. The comprehensive information will help pave the way to an understanding of the different mechanisms involved in producing the unique fatty acid profiles of palm mesocarp and kernel oils.

## Conclusions

An integrated gene prediction pipeline was developed, enabling annotation of the African oil palm genome, and deriving a set of 26,059 high quality and thoroughly validated gene models. BUSCO analysis showed that our high-quality gene models contain at least 90% of the known conserved orthologs in eukaryotes, making our gene prediction collection the most reliable annotation of the oil palm genome. With the results, we conducted an in-depth analysis of several important gene categories: intronless, resistance and FA biosynthesis. The prevalence of these groups was similar across several plant genomes, including those of *A. thaliana*, *Z. mays*, *O. sativa, S. bicolor*, *G. max* and *R. communis*. Coding regions of the oil palm genome have a characteristic broad distribution of GC_3_, with a heavy tail extending to high GC_3_ values that contain many stress-related and intronless genes. GC_3_-rich genes in oil palm are significantly over-represented in the following GOslim process categories: responses to abiotic stimulus, responses to endogenous stimulus, RNA translation, and responses to stress. We found approximately one-seventh of the oil palm genes identified to be intronless. Two hundred ten R genes grouped in six classes based on their protein domain structures were also identified. Lipid-, especially FA-related genes, are of interest in oil palm where, in addition to their roles in specifying oil yield and quality, also contribute to the plant organization and are important for biotic and abiotic stress signaling. We identified 42 key genes involved in oil palm FA biosynthesis, which will be especially useful for oil palm breeders.

The results from our study will facilitate understanding of the plant genome organization, and be an important resource for further comparative and evolutionary analysis. The study of oil palm genes will facilitate future advances in the regulation of gene function in the crop, and provide a theoretical foundation for marker-assisted breeding for increased oil yield and elevated oleic and other valuable fatty acids.

## Reviewers’ comments

### Reviewer’ report 1: Alexander Kel, Genexplain, Germany

#### Reviewer comments

In this paper, the authors have successfully annotated the oil palm genome with high quality annotation of over 26 thousand genes. An important novelty of the approach is application of two independent gene prediction pipelines Fgenesh++ and Seqping that are best available, at least for plant genomes. The gene prediction is combined with many additional lines of evidences, applying really a big number of various tools, that makes it a top quality genome annotation initiative. Very important is that the authors combined the pure computational efforts with the experimental transcriptomics analysis (using RNA-seq) which helped them to perform better gene annotation and also gives additional possibility for functional interpretation of the results. In summary, I am recommending this manuscript for rapid publication, which will provide the community with a new rich resource for analysis of these very important genome.

1) The own tissue-specific RNA-sequencing data (from MPOB) used in the paper should be better described. Ideally in a separate section.

Author’s response: *We thank the reviewer for the kind suggestion and have added the list of the RNA-sequencing libraries in Additional file* 1 *.*

2) Rules of integration between results of the two pipelines used should be also a bit better described. The Table 2 is a little bit confusing. Perhaps an example with overlapping gene models coming from two different tools could be helpful for the reader.

Author’s response: *To merge pipelines, we looked at clusters of genes with continuous overlap within the cluster at different percentages of the length. Each gene in the cluster overlaps with at least one other gene from the cluster at a given overlap threshold (single linkage approach). ORF predictions with < 300 nucleotides were excluded. We tested different overlap thresholds from 60% to 95% in 5% increments, as shown in Fig.* 2 *. Gene models from the same strand predicted from the two pipelines are considered to belong to the same locus if the gene models within the locus overlap at the selected threshold with at least one other gene in the locus. In a locus, gene models can overlap at different regions as shown in* Additional file 3 *: Figure S1a. Gene models that do not meet the overlap threshold will form different sets of genes (*Additional file 3 *: Figure S1B). Overlap of 85% was selected as the best threshold, as the rate of increase in the number of single gene loci was higher after this threshold level. The representative gene model for each locus was selected based on the gene model with the lowest E-value comparison to RefSeq in the respective locus. The details of how the representative gene models are selected are described in Methods section (Line 246-263).*

3) Concerning the intron-less genes (IG). I think that more explanations are needed to argue that the IG genes are actually “working” genes in genome, but not possible pseudo-genes. As we can see from the Table 1, only a fraction of the predicted genes has got evidence from the transcriptomics and RefSeq that they are actually transcribed. What is the fraction of IG genes has got such evidence?

Author’s response: *The IG genes that were characterized in the manuscript originated from the 26,059 representative genes models with both RefSeq and oil palm transcriptome evidence. They are from the “high-confidence” subset of all genes presented in the* Fig. 1 *. This is also mentioned in Line 358-360. Table 1 was changed to a flow chart (*Fig. 1 *) to improve clarity.*

### Reviewer’s report 2: Igor Rogozin, NIH, USA

#### Reviewer comments

The paper describes a new annotation of 26,059 oil palm genes using two independent gene-prediction pipelines, Fgenesh++ and Seqping. The authors identified 42 key genes involved in FA biosynthesis in oil palm. For three of these genes, namely EgFABF, EgFABH and EgFAD3, recent duplication events were detected.

1) I would define GC3 in the Abstract.

Author’s response: *The description of GC*_*3*_ *has been added to the Abstract (Line 109).*

2) "with a heavy tail of high GC_3_ regions harboring many intronless and stress-related genes..." Is this result supported by statistical test(s)?

Author’s response: *Additional text had been added in the GC*_*3*_ *(Line 442-443) and GO analysis (Line 415-423) sections to address this issue. 36% of the intronless genes were GC*_*3*_ *-rich while GO analysis showed that there were higher representations of stress-related genes in the GC*_*3*_ *-rich gene set as compared to all the oil palm genes.*

3) "Our analysis indicates that de novo FA biosynthesis in the oil palm mesocarp and kernel is driven primarily by EgFAB2_1." I am not sure that the authors have enough support for this statement. Maybe I missed something.

Author’s response: *We agree with the reviewer and have removed the statement. In the results section, the gene is listed as “the dominant copy of the FAB2 gene, and largely responsible for conversion of C18:0-ACP to C18:1-ACP in de novo FA biosynthesis in the oil palm mesocarp and kernel” as it has the highest expression in both tissues. We thank the reviewer for his comments.*

4) Conclusions in the Abstract looks too general: "...while providing theoretical foundation for marker-assisted breeding of this globally important crop". The authors may try to make this section more specific.

Author’s response: *We are grateful to the reviewer for his recommendations and have edited the Conclusions section in the Abstract to better reflect the manuscript.*

### Reviewer’s report 3: Vladimir A. Kuznetsov, Bioinformatics Institute, Singapore

#### Reviewer comments

In this study, the authors develop an integrated gene-finding framework and applied it to identify high quality oil palm gene models using the pisifera scaffold assembly and combining mapping pipelines. The best gene model for each locus was selected to establish a representative “high confidence” gene set. This paper provides identification and characterization of the “high confidence” set of 26,059 oil palm genes that have transcriptome and RefSeq support, and is supported by bioinformatics analysis of the genes. The study includes comparative genomics and regular bioinformatics analyses, statistical tests and new database. It is a well- designed and interesting study. However, several important statements, results and their interpretation have to be clarified and improved.

1) I suggest to revised the Abstract. Background. Replace a common introduction sentences “Emergence of rapid and inexpensive DNA sequencing technology has led to an avalanche of data waiting to be transformed into valuable insight about genome organization and function. A typical starting point for genome analysis is, customarily, annotation” onto more specific scientific problem(s) in the oil palm genome biology (e.g., accurate gene annotation) and the alignment of the methods and results to the palm oil industry needs (oil yields and quality) and/or economic efficiency of the industry. “This paper presents a study of the oil palm genome, including comparative genomics analysis, along with the development of the relevant database and < bioinformatics> tools.” Method section information is not present. Results: The sentence “Our analysis indicates that de novo FA biosynthesis in the oil palm mesocarp and kernel is driven primarily by EgFAB2_1.” is too strong for a bioinformatics paper. Conclusions. The conclusion is week and is not specific. The phrase “The study of oil palm genome will facilitate further understanding of its genetic regulation” is not a main result of this study. The phrase “providing theoretical foundation” is not correct in the context of the aims of this study.

Author’s response: *We agree with the reviewer and have edited the Abstract. The Background section had been changed to provide some information on the oil palm and the reasons for the study. Although we do not have a Methods section, which is in line with the requirements of the journal, the methods used had been incorporated into the Results section. We agree with the reviewers that the statement for EgFAB2_1 is too strong and have removed it. The conclusions have also been edited to better reflect the manuscript.*

2) Information about database should be included in the Method/Result sections.

Author’s response: *Information on how to access the database is available in the Declaration section. We have also added this information in the Abstract section. Information on the database has also been added to the Results section (Line 360-364) and* Additional file 4 *.*

3) Three-four major results should be summarized in the conclusion.

Author’s response: *We thank the reviewer for the constructive comment and have edited the Conclusions section in the Abstract to better reflect the manuscript.*

4) Introduction Goals: You should better specify a goal and problem’s vision. For example, the objectives of the programme complex and the database may be: 1. To develop a high standard gene reference/annotation system for the oil palm genome analysis. 2. To map the genes and regulatory DNA signals/sequences associated with important agronomic traits. 3. To develop and use the genome information to solve the disease and stress resistant palms with enhanced productivity.

Author’s response: *The final paragraph of the Introduction section has been edited to better reflect the goals of the project.*

5) Methods The workflow for the gene prediction method and the data analysis should be included.

Author’s response: *We have improved the Methods section to provide more details of the processes used and added the flowchart of the pipeline. The details of the gene prediction are described in the Methods section under the headers “Fgenesh++ Gene Prediction” and “Seqping Gene Prediction”. The processes to integrate the gene models from both pipelines are described in the “Integration of Fgenesh++ and Seqping Gene Predictions” section.*

6) Database. In fact, you did not use your DB to support the results. The DB should be more important part of your work, to be described and actively used in the study. You may provide the figure(s) showing Web interface and add user-friendly help/comment information. A few examples (figure(s)) of the useful tracks supporting the major statements (known important and novel genes, joint tracks of the gene models and transcription data and key regulatory signals etc.) could make this study more interesting and attractive.

Author’s response: *The database, PalmXplore is an integrated database system that allows researchers to search, retrieve and browse the oil palm gene information and associated functional annotations using a convenient interface and fast database on the back-end. It was developed as a tool for researchers to easily search and access the results of this study. The URL of the database is available in the Abstract and Declaration section. We have also added additional information on the database in the Results section (Line 360-364) and* Additional file 4 *.*

7) pp.8-9 Reproducibility and availability issues: Information about the “high confidence” gene set, chromosome coordinates of these genes should be available in (new) master table. Information about gene structure and annotation shown for the intronless, two and more exons genes could be useful for future studies. p.11 “all genes by their GC3 content and designated the top 10% (2,605 ORFs) as GC3-rich (GC3≥0.75), and the bottom 10% as GC3-poor (GC3≤0.37).” Reproducibility and accessibility of main data/results is an important issue. Could you please include in (new) master table data for 2605 ORFs with explicit presentation of the GC3-rich and GC3-poor, and GC-skew characteristics of the genes/transcript isoforms, specifying the intron-less and multiple exon genes, UTRs, exon and intron locations? The data base should be also updated accordingly. The including help file, summary statistics and a few examples will be much appreciated.

Author’s response: *We have included a table in* Additional file 1 *. The location and structure of the genes is available in the PalmXplore database. The URL of the database has been included in the manuscript.*

8) p. 11 and Fig. 3. “Despite the relatively small number of the GC3-rich genes in the oil palm genome, there are characteristic patterns of positional gradients (Fig. 3c and d) near the predicted start of translation…”. Fig. 3c and d does not provide information about the frequency distribution of GC3 in upstream or downstream regions of the transcription start site (TSS). You should construct that frequency distribution function using the GC-skew sequence data for TSS of the annotated genes of interest.

Author’s response: *GC3 is a frequency of cytosines and guanines in the third position of codon. It is therefore only used to define the cytosines and guanines levels of the coding regions. The present manuscript focuses on the generation, characterization and annotation of high quality gene models or the genic regions of the oil palm genome. Although we agree that characterization of the promoter region is important, it is not within the present scope of the manuscript. We are currently working on the best method to predict the TSS and promoter regions accurately*.

9) p.11 and Fig. 3d CG3 skew gradient along the open reading frames of GC3-rich and -poor genes. Axis Y shows the CG-skew score calculated by Eq. CG-skew = (C-G)/(C + G). However, in the main text this formula was not introduced and discussed; instead, CG3-skew = (C3-G3)/(C3 + G3) was introduced and discussed, where the C3 and the G3 were not defined. Please explain and make appropriate corrections.

Author’s response: *We thank the reviewer for the comment. There was a typo error in the y-axis of* Fig. 3 *(now* Fig. 4 *) and it has been corrected.* Fig. 3d *(now* Fig. 4d *) now shows CG3-skew. We have also added an explanation in the figure legend.*

10) p.11 Analysis of the GC contents, GC-skew characteristics in exons are not enough to characterise the regulatory signals and biological complexity of the genes at the genome and transcriptome scales. For the identification of gene regulatory signals, specifically for the transcription initiation and termination, it is important to analysis the GC-skew regions and the G-rich clusters in the proximal promoter regions of a gene, gene body, downstream gene region (not only the exons). These kinds of signals can provide specific gene expression regulation often associated with the transcriptional R-loop formation sequences. It has been shown that the R-loop formation structures (RLFS) could be reliably identified/predict by QmRRFS tool [Wongsurawat et al., NAR, 2012; Jenjaroenpun et al., NAR, 20,015], predicting the RLFS sequences within the proximal gene regions and in gene body at accuracy 90–92%. Mapping RLFS data, you could increase power and the specificity of the gene models. This analysis could provide the links of the gene models with key regulatory signals related to initiation of transcription, polymerase pausing sites, alternative starts and splice variances, open chromatin regions, disease critical regions etc. All these genome signals are strongly associated with RLFS locations [Wongsurawat et al., NAR, 2012; Jenjaroenpun et al., NAR, 20,015, Ginno et al., Genome Res., 2013, Sanz et al., Molecular Cell, 2016]. The RLFS analysis may make this study more interesting, novel and biologically important.

Author’s response: *This is an excellent suggestion. We used QmRRFS to find R-loop forming sequences (RLFS) in the region [ATG-2000, ATG + 40] of each gene* [153–156]*. We found that the region immediately upstream from ATG, [ATG-200, ATG] is significantly enriched for RLFS (p-value ~ 0.0). However, the study of R-loops, which are essential for transcriptional processes, is not part of the present study that focuses on the coding regions, and will be part of the next study. Also, the oil palm genome currently does not have a collection of full-length cDNA sequences. Once we are able to predict the oil palm TSS accurately, we will analyze CG skews, R-loops and other features. These analyses will be presented in a separate manuscript once the analysis is complete.*

11) p. 11 Gene ontology analysis shows that many of the GC3-rich genes are stress-related, while many of the GC3-poor genes have housekeeping functions (see GO annotation in Additional file 2: Table S2). However, Table 2 shows more diverse (and actually interesting) results, which also suggest a weakness of authors’ statement. Indeed, sorting out the GO categories in Additional file 2: Table S2 by the score S = (CG3-rich –CG3-poor)/(CG3-rich + CG-poor) at smallest cut-off value of the score equals |0.2|, we observed, that 10 most strong terms (oxygen binding, structural molecule activity, secondary metabolic process, translation, sequence-specific DNA binding transcription factor, response to abiotic stimulus, cell growth, response to endogenous stimulus (last ranked term)) are following the condition S > 0.2 (CG3-rich). Furthermore, the 17 GO terms (regulation of gene expression and epigenetic, motor activity, RNA binding, nucleotide binding, nuclease activity, lipid binding, kinase activity, nucleic acid binding, chromatin binding, translation factor activity, nucleic acid binding, signal transducer activity, protein metabolic process, catabolic process, hydrolase activity, embryo development, cell cycle, response to extracellular stimulus (last ranked term)) are following the condition S < −0.2 (CG3-poor). I propose that the more balanced and complete analysis, interpretation and discussion of the GO enrichment data analysis will be carried out.

Author’s response: *We have calculated the enrichment statistics:(#GC3-rich-#GC3-poor)/Total number of genes, (#GC3-rich-#GC3-poor)/(#GC3rich + #GC3-poor), and also computed the chi-squared statistics. The results are shown in the GO enrichment table in* Additional file 1 *.*

12) Additional file 2: Table S8 Could you please explain and discuss the observed differences between percentage intronless (PI) genes in GC3 -rich genes belonging to the same GO branch “growth” (PI = 19%), “cell growth” (PI = 13%), “cell cycles” (PI = 6) Table 8)? How many of the “cell cycle” genes are included in “growth” and “cell growth” categories? How many of the “cell cycle genes” are unique?

Author’s response: *There are no genes that belong to all three categories (“growth”, “cell growth”, and “cell cycle”). However, there are genes in the intersection of two categories. The numbers of annotated genes that fall into the three categories are as follows:* 
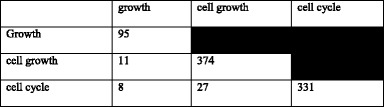


*The annotations of the INTRONLESS genes are listed below:* 
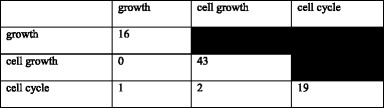


13) Intronless genes analysis It may be important and interesting to carry out meta-gene analysis providing the density function of GC-skew and RLFS sequence occurrences (count of the number of the sequences in a given nucleotide location) within TSS vicinity for the intron-less genes and the genes having multiple introns. It may provide new knowledge about structural and regulatory roles of the RLFS and GC-skew sequences in the intronless genes and the multi-exon genes in the oil palm genome.

Author’s response: *This analysis will be conducted in our next paper dedicated to TSS prediction and analysis of regulatory sequences.*

## Additional files

Additional file 1: Includes information of oil palm RNA-seq data, annotation of IG, R genes and FAB genes, GO and GC3. (XLSX 8028 kb)

Additional file 2: Supplementary Tables. (DOCX 44 kb)

Additional file 3: Supplementary Figures. (DOCX 4776 kb)

Additional file 4: Additional file 4 provides screenshots of PalmXplore. (DOCX 962 kb)

## Abbreviations

ACCase: Acetyl-CoA carboxylase
ACP: Acyl carrier protein
Avr: Avirulence
CC: Coiled-coil
CDS: Coding sequence
CNL: CC-NBS-LRR
FA: Fatty acid
FAB2: Stearoyl-ACP desaturase
FABF: β-ketoacyl-ACP synthase II
FAD2: Oleoyl-phosphatidylcholine desaturase
FAD3: Linoleoyl-phosphatidylcholine desaturase
FATA: Oleoyl-ACP thioesterase
FATB: Acyl-ACP thioesterase
GO: Gene ontology
IG: Intronless gene
LRR: Leucine-rich repeat
NBS: Nucleotide binding site
R: Resistance
STK: Serine/threonine protein kinase
TNL: Toll/interleukin-1 NBS-LRR
