# Supplementary material for: Evidence-based gene models for structural and functional annotations of the oil palm genome
Source: Biol Direct. 2017 Sep 8;12:21. doi: 10.1186/s13062-017-0191-4 (PMC5591544; doi:10.1186/s13062-017-0191-4)
Supplement: Supplementary file 2 — Supplementary Tables. (DOCX 44 kb) [file 13062_2017_191_MOESM2_ESM.docx]

Additional file 2

Table S1: Number of loci at different overlap thresholds between Fgenesh++ and Seqping gene models

| Number of isoforms in locus | Number of loci at different overlap threshold | | | | | | | |
| --- | --- | --- | --- | --- | --- | --- | --- | --- |
|  | 60% | 65% | 70% | 75% | 80% | **85%** | 90% | 95% |
| 1 | 17312 | 17402 | 17518 | 17656 | 17825 | **18047** | 18349 | 18948 |
| 2 | 12881 | 12873 | 12837 | 12788 | 12722 | **12636** | 12531 | 12290 |
| 3 | 731 | 709 | 696 | 683 | 672 | **657** | 631 | 603 |
| 4 | 63 | 61 | 61 | 61 | 60 | **60** | 59 | 52 |
| 5 | 14 | 14 | 13 | 14 | 14 | **13** | 11 | 10 |
| 6 | 1 | 1 | 1 | 0 | 0 | **0** | 0 | 0 |
| all loci | 31002 | 31060 | 31126 | 31202 | 31293 | **31413** | 31581 | 31903 |

Table S2: BUSCO analysis of three oil palm genome annotations

| BUSCO category | Pisifera annotation,  26,059 gene models | Dura annotation  (Jin et al. 2016),  36,105 gene models | EG5 annotation  (Singh et al. 2013),  30,752 gene models |
| --- | --- | --- | --- |
| Complete BUSCOs (C), including: | 1,170 | 765 | 599 |
| - Complete and single-copy BUSCOs (S) | 1,031 | 687 | 548 |
| - Complete and duplicated BUSCOs (D) | 139 | 78 | 51 |
| Fragmented BUSCOs (F) | 65 | 145 | 270 |
| Missing BUSCOs (M) | 205 | 530 | 571 |
| Total BUSCOs | 1,440 | 1,440 | 1,440 |

Table S3a: Relationship between GO process annotation and GC_3_ content of oil palm genes

| GO Process | Total number of genes | GC_3_ poor^#^ | GC_3_ rich^#^ |
| --- | --- | --- | --- |
| response to abiotic stimulus | 1996 | 164 | 299 |
| translation | 1082 | 43 | 104 |
| response to endogenous stimulus | 1372 | 138 | 204 |
| secondary metabolic process | 364 | 12 | 54 |
| biological_process | 7812 | 788 | 891 |
| cell growth | 423 | 41 | 65 |
| biosynthetic process | 4692 | 464 | 538 |
| response to stress | 2905 | 255 | 324 |
| response to biotic stimulus | 928 | 87 | 106 |
| cellular homeostasis | 231 | 24 | 30 |
| photosynthesis | 168 | 16 | 22 |
| anatomical structure morphogenesis | 892 | 89 | 101 |
| transport | 2359 | 224 | 228 |
| pollination | 156 | 15 | 18 |
| tropism | 81 | 10 | 10 |
| lipid metabolic process | 933 | 91 | 89 |
| cell communication | 178 | 20 | 16 |
| response to external stimulus | 105 | 12 | 8 |
| generation of precursor metabolites and energy | 322 | 38 | 30 |
| cell differentiation | 571 | 65 | 62 |
| carbohydrate metabolic process | 1107 | 116 | 97 |
| response to extracellular stimulus | 191 | 25 | 15 |
| multicellular organismal development | 1560 | 175 | 169 |
| cell death | 253 | 25 | 16 |
| flower development | 526 | 59 | 39 |
| signal transduction | 1143 | 134 | 129 |
| cell cycle | 371 | 52 | 30 |
| post-embryonic development | 1213 | 154 | 105 |
| nucleobase, nucleoside, nucleotide and nucleic aci | 3795 | 455 | 377 |
| embryo development | 661 | 93 | 48 |
| reproduction | 967 | 135 | 87 |
| cellular process | 6709 | 749 | 589 |
| growth | 101 | 23 | 17 |
| protein modification process | 1884 | 244 | 147 |
| protein metabolic process | 915 | 116 | 48 |
| cellular component organization | 1564 | 234 | 145 |
| catabolic process | 1510 | 213 | 92 |
| regulation of gene expression, epigenetic | 142 | 40 | 3 |

* GO categories with at least 20 genes are listed.

^#^ GC_3_-poor and -rich genes are selected as bottom and top 10% genes ranked by GC_3_ composition; 2606 genes in each category.

Table S3b: Relationship between GO function annotation and GC_3_ content of oil palm genes

| GO Function | Total number of genes | GC_3_ poor^#^ | GC_3_ rich^#^ |
| --- | --- | --- | --- |
| structural molecule activity | 921 | 21 | 106 |
| sequence-specific DNA binding transcription factor | 1824 | 131 | 258 |
| oxygen binding | 105 | 3 | 24 |
| DNA binding | 1552 | 174 | 188 |
| enzyme regulator activity | 272 | 33 | 41 |
| transferase activity | 1739 | 171 | 203 |
| transporter activity | 1471 | 122 | 154 |
| catalytic activity | 3221 | 324 | 336 |
| receptor activity | 101 | 11 | 11 |
| carbohydrate binding | 124 | 11 | 7 |
| protein binding | 3280 | 365 | 325 |
| translation factor activity, nucleic acid binding | 163 | 21 | 8 |
| signal transducer activity | 205 | 25 | 10 |
| lipid binding | 209 | 32 | 11 |
| DNA metabolic process | 470 | 69 | 60 |
| chromatin binding | 52 | 14 | 5 |
| motor activity | 97 | 20 | 2 |
| nuclease activity | 207 | 40 | 11 |
| binding | 3407 | 449 | 347 |
| kinase activity | 996 | 151 | 52 |
| nucleic acid binding | 697 | 122 | 43 |
| hydrolase activity | 2613 | 331 | 169 |
| RNA binding | 685 | 118 | 25 |
| nucleotide binding | 1849 | 305 | 77 |

* GO categories with at least 20 genes are listed.

^#^ GC_3_-poor and -rich genes are selected as bottom and top 10% of genes ranked by GC_3_ composition; 2606 genes in each category.

Table S4: Multinomial model of oil palm gene’s nucleotide usage

| Class | A | C | G | T |
| --- | --- | --- | --- | --- |
| Rich | 0.181253 | 0.334665 | 0.301814 | 0.182268 |
| Poor | 0.293708 | 0.193447 | 0.235379 | 0.277467 |

Table S5: Multinomial position-specific model of oil palm gene’s nucleotide usage

| Class | Position | A | C | G | T |
| --- | --- | --- | --- | --- | --- |
| Rich | 1 | 0.212395 | 0.255631 | 0.36181 | 0.170163 |
|  | 2 | 0.253268 | 0.275597 | 0.201407 | 0.269727 |
|  | 3 | 0.067188 | 0.488286 | 0.345706 | 0.098819 |
| Poor | 1 | 0.281471 | 0.1967 | 0.326162 | 0.195667 |
|  | 2 | 0.319652 | 0.238278 | 0.177159 | 0.26491 |
|  | 3 | 0.281411 | 0.144925 | 0.200389 | 0.373275 |

Table S6: First order three periodic Markov Chain model for nucleotide usage of GC_3_-rich genes

| Position | Previous | A | C | G | T |
| --- | --- | --- | --- | --- | --- |
| 1 | A | 0.211883 | 0.193801 | 0.379371 | 0.214945 |
| 1 | C | 0.206733 | 0.274664 | 0.332857 | 0.185745 |
| 1 | G | 0.243725 | 0.228174 | 0.391242 | 0.13686 |
| 1 | T | 0.131988 | 0.297704 | 0.390492 | 0.179816 |
| 2 | A | 0.325736 | 0.219079 | 0.179705 | 0.275467 |
| 2 | C | 0.198185 | 0.25031 | 0.207227 | 0.344277 |
| 2 | G | 0.299278 | 0.283979 | 0.217995 | 0.198743 |
| 2 | T | 0.147727 | 0.3663 | 0.184483 | 0.30149 |
| 3 | A | 0.077361 | 0.391751 | 0.414435 | 0.116453 |
| 3 | C | 0.070179 | 0.526497 | 0.301946 | 0.101378 |
| 3 | G | 0.08692 | 0.459979 | 0.368366 | 0.084735 |
| 3 | T | 0.039847 | 0.561023 | 0.308967 | 0.090163 |

Table S7: First order three periodic Markov Chain model for nucleotide usage of GC_3_-poor genes

| Position | Previous | A | C | G | T |
| --- | --- | --- | --- | --- | --- |
| 1 | A | 0.307091 | 0.170316 | 0.320025 | 0.202568 |
| 1 | C | 0.432052 | 0.219156 | 0.098793 | 0.249999 |
| 1 | G | 0.31151 | 0.216113 | 0.314642 | 0.157735 |
| 1 | T | 0.187714 | 0.197384 | 0.425098 | 0.189805 |
| 2 | A | 0.361696 | 0.166196 | 0.214317 | 0.257791 |
| 2 | C | 0.341448 | 0.257338 | 0.110473 | 0.290737 |
| 2 | G | 0.385121 | 0.219078 | 0.20221 | 0.193591 |
| 2 | T | 0.128128 | 0.354815 | 0.148988 | 0.368069 |
| 3 | A | 0.285635 | 0.123713 | 0.238971 | 0.351681 |
| 3 | C | 0.391244 | 0.135954 | 0.054697 | 0.418102 |
| 3 | G | 0.259284 | 0.186466 | 0.225481 | 0.328769 |
| 3 | T | 0.192319 | 0.150809 | 0.268098 | 0.388774 |

Table S8: Sequence lengths of 2606 GC_3_-rich and -poor genes

| Sequence type | All genes | GC_3_-rich, GC_3_≥0.75286 | GC_3_-poor, GC_3_≤0.373239 |
| --- | --- | --- | --- |
| GENE | 7,147 | 1,893 | 13,130 |
| ORF | 1,237 | 928 | 1,795 |

Table S9: Relationship between gene ontology classification, GC_3_ and prevalence of intronless genes

| GOSLIM_TERM | Number of genes | GC_3_ | Number of intronless genes | GC_3_ intronless | Percent intronless |
| --- | --- | --- | --- | --- | --- |
| cell-cell signaling | 29 | 0.57 | 9 | 0.80 | 31% |
| pollen-pistil interaction | 41 | 0.46 | 8 | 0.52 | 20% |
| growth | 101 | 0.53 | 19 | 0.81 | 19% |
| translation | 1082 | 0.59 | 177 | 0.70 | 16% |
| response to endogenous stimulus | 1372 | 0.56 | 210 | 0.74 | 15% |
| secondary metabolic process | 364 | 0.60 | 54 | 0.71 | 15% |
| response to stress | 2905 | 0.53 | 423 | 0.66 | 15% |
| response to abiotic stimulus | 1996 | 0.56 | 280 | 0.70 | 14% |
| response to biotic stimulus | 928 | 0.54 | 126 | 0.68 | 14% |
| nucleobase, nucleoside, nucleotide and nucleic aci | 3795 | 0.52 | 498 | 0.66 | 13% |
| biosynthetic process | 4692 | 0.54 | 615 | 0.70 | 13% |
| cell death | 253 | 0.52 | 32 | 0.65 | 13% |
| cell differentiation | 571 | 0.53 | 72 | 0.72 | 13% |
| cell growth | 423 | 0.55 | 53 | 0.72 | 13% |
| photosynthesis | 168 | 0.53 | 21 | 0.75 | 13% |
| signal transduction | 1143 | 0.53 | 140 | 0.70 | 12% |
| protein modification process | 1884 | 0.50 | 225 | 0.65 | 12% |
| cellular homeostasis | 231 | 0.55 | 26 | 0.67 | 11% |
| anatomical structure morphogenesis | 892 | 0.53 | 100 | 0.70 | 11% |
| multicellular organismal development | 1560 | 0.53 | 168 | 0.69 | 11% |
| metabolic process | 6114 | 0.52 | 643 | 0.69 | 11% |
| DNA metabolic process | 470 | 0.50 | 47 | 0.76 | 10% |
| ripening | 10 | 0.57 | 1 | 0.52 | 10% |
| transport | 2359 | 0.52 | 233 | 0.68 | 10% |
| reproduction | 967 | 0.51 | 94 | 0.66 | 10% |
| cellular component organization | 1564 | 0.50 | 143 | 0.71 | 9% |
| post-embryonic development | 1213 | 0.51 | 108 | 0.65 | 9% |
| cellular process | 6709 | 0.52 | 597 | 0.69 | 9% |
| generation of precursor metabolites and energy | 322 | 0.51 | 28 | 0.74 | 9% |
| embryo development | 661 | 0.51 | 57 | 0.64 | 9% |
| pollination | 156 | 0.52 | 13 | 0.69 | 8% |
| flower development | 526 | 0.52 | 43 | 0.67 | 8% |
| abscission | 25 | 0.57 | 2 | 0.58 | 8% |
| protein metabolic process | 915 | 0.50 | 71 | 0.62 | 8% |
| response to extracellular stimulus | 191 | 0.50 | 14 | 0.69 | 7% |
| lipid metabolic process | 933 | 0.52 | 67 | 0.78 | 7% |
| carbohydrate metabolic process | 1107 | 0.52 | 78 | 0.73 | 7% |
| response to external stimulus | 105 | 0.52 | 7 | 0.74 | 7% |
| cell cycle | 371 | 0.48 | 23 | 0.68 | 6% |
| catabolic process | 1510 | 0.49 | 91 | 0.67 | 6% |
| cell communication | 178 | 0.51 | 9 | 0.73 | 5% |
| regulation of gene expression, epigenetic | 142 | 0.43 | 5 | 0.51 | 4% |
| tropism | 81 | 0.55 | 2 | 0.62 | 2% |
| behavior | 6 | 0.44 | 0 | NA | 0% |

Table S10: Length and number of intronless genes in different genomes

| Genome Name | Taxonomy | Genome Size  (Mbp) | Number of Intronless Genes | Number of Genes |
| --- | --- | --- | --- | --- |
| *Elaeis guineensis* | Monocot | 1,800 | 3,658 (14%) | 26,059 |
| *Zea mays* | Monocot | 2,300 | 14,623 (37%) | 39,469 |
| *Sorghum bicolor* | Monocot | 730 | 6,321 (23%) | 27,608 |
| *Oryza sativa* | Monocot | 500 | 14,958 (26%) | 57,840 |
| *Volvox carteri* | Algae | 140 | 2,419 (16%) | 15,285 |
| *Arabidopsis thaliana* | Dicot | 135 | 7,368 (20%) | 37,513 |
