# Supplementary material for: Evidence-based gene models for structural and functional annotations of the oil palm genome
Source: Biol Direct. 2017 Sep 8;12:21. doi: 10.1186/s13062-017-0191-4 (PMC5591544; doi:10.1186/s13062-017-0191-4)
Supplement: Supplementary file 3 — Supplementary Figures. (DOCX 4776 kb) [file 13062_2017_191_MOESM3_ESM.docx]

Addiitonal file 3





Figure S1: Example of the gene models with overlapping regions. (a) Single gene model from Seqping is integrated with multiple short gene models from the FGENESH++ pipeline. (b) Continuous cluster of overlapping genes from two pipelines using the single linkage approach. Gene models that do not meet the ≥85% overlap threshold will form 2 different locus (2 boxes). Gene models in green colour are selected as the representative gene model for the locus.





Figure S2: Multiple sequence alignment of Kinase class R genes. Autophosphorylation sites in the activation domain indicated in black circles. Black box shows Thr to Gly mutation in autophosphorylation site of oil palm R genes.





Figure S3: Physical clusters of candidate R genes in oil palm chromosomes.





Figure S4: Multiple sequence alignment of malonyl-CoA:ACP transacylase (FABD) homologues. The five conserved residues: Gln(Q), Ser(S), Arg(R), His(H) and Gln(Q) are shaded in black and their respective positions indicated. The boxes show the conserved G(L/H)SLG motif and its corresponding PFAM HMMLogo. GenBank accession numbers: Eg, EgFABD; Ah, **EU823322**; Gm, **ABB85235**; At1, **AAM14913**; At2, **AAM64515**; Bn, **CAB45522**; Pf, **AAG43518**; Ca, **ACF17665**; Os, **ABF95452**; Ec, **1MLA**. Abbreviations: Eg, *Elaies guineensis*; Ah, *Arachis hypogaea*; Gm, *Glycine max*; At, *Arabidopsis thaliana*; Bn, *Brassica napus*; Pf, *Perilla frustescens*; Ca, *Capsicum annuum*; Os, *Oryza sativa*; Ec, E*scherichia. coli*. (*) fully conserved residues; (:) conservation between groups of strongly similar properties, (.) conservation between groups of weakly similar properties.





Figure S5: Multiple sequence alignment of β-ketoacyl-ACP synthase I (FABB) homologues. The three conserved residues: Cys(C), His(H) and His(H) are shaded in black and their respective positions indicated. GenBank accession numbers: Eg1, EgFABB_1; Eg2, EgFABB_2; Eg3, EgFABB_3; Eg4, EgFABB_4; Ah, **EU823325**; Gm1, **AAF61730**; Gm2, **AAF61731**; At1, **AAC49118**; At2, **AAM65396**; Pf, **AAC04691**; Rc, **AAA33873**; Jc, **ABJ90468**; Os, **BAD35225**; Hv, **AAA32968**; Ec, **AAC67304**. Abbreviations: Eg, *E. guineensis*; Ah, *A. hypogaea*; Gm, *G. max*; At, *A. thaliana*; Pf, *P. frustescens*; Rc, *Ricinus communis*; Jc, *Jatropha curcas*; Os, *O. sativa*; Hv, *Hevea vulgare*; Ec, *E. coli*. (*) fully conserved residues; (:) conservation between groups of strongly similar properties, (.) conservation between groups of weakly similar properties.





Figure S6: Multiple sequence alignment of β-ketoacyl-ACP synthase II (FABF) homologues. The three conserved residues Cys(C), His(H) and His(H) are shaded in black and their respective positions indicated. GenBank accession numbers: Eg1, EgFABF_1; Eg2, EgFABF_2; Eg3, EgFABF_3; Ah, **EU823327**; Gm1, **AAW88762**; Gm2, **AAW88763**; Gm3, **AAF61737**; At1, **AAK69603**; At2, **AAL91174**; Pf, **AAC04692**; Rc, **AAA33872**; Jc, **ABJ90469**; Os, **BAC79989**; Hv1, **CAA84022**; Hv2, **CAA84023**; Ec, **CAA84431**. Abbreviations: Eg, *E. guineensis*; Ah, *A. hypogaea*; Gm, *G. max*; At, *A. thaliana*; Pf, *P. frutescens*; Rc, *R. communis*; Jc *J. curcas*; Os; *O. sativa*; Hv, *H. vulgare*; Ec, *E. coli*. (*) fully conserved residues; (:) conservation between groups of strongly similar properties, (.) conservation between groups of weakly similar properties.





Figure S7: Multiple sequence alignment of β-ketoacyl-ACP synthase III (FABH) homologues. The four conserved residues Cys(C), His(H), Arg(R), and Asn(N) are shaded in black and their respective positions indicated. The boxes show the conserved GNTSAAS motif and its corresponding PFAM HMMLogo. GenBank accession numbers: Eg1, EgFABH_1; Eg2, EgFABH_2; Ah, **EU823328**; Gm, **AAF70509**; At1, **AAA61348**; At2, **CAA72385**; Ca1, **ACF17661**; Ca2, **ACF17662**; Cw1, **AAA97533**, Cw1, **AAA97533**; Cw2, **AAA97534**; Ch1, **AAF61398**; Ch2, **AAF61399**; Pf1, **AAC04693**; Pf2, **AAC04694**; Aa, **AAB61310**; Ps, **CAC08184**; Rc, **ABR12417**; Jc, **ABJ90470**; So, **CAA80452**; Ha, **ABP93352**; Eg, **ABE73469**; Eo, **ABE73470**; Ec, **AAA23749**. Abbreviations: Eg, *E. guineensis*; Ah, *A. hypogaea*; Gm, *G. max*; At, *A. thaliana*; Ca, *C. annuum*; Cw, *Colpothrinax* wrightii; Ch, C*uphea. hookeriana*; Pf, *P. frustescens*: Aa, *Allium ampeloprasum*; Ps, *Pisum sativum*; Rc, *R. communis*; Jc, *J. curcas*; So, *Spinacia oleracea*; Ha, *Helianthus annuus*; Eo, *E. oleifera*; Ec, *E. coli*. (*) fully conserved residues; (:) conservation between groups of strongly similar properties, (.) conservation between groups of weakly similar properties.





Figure S8: Multiple sequence alignment of β-ketoacyl-ACP reductase (FABG) homologues. The five conserved residues Lys(K), Ser(S), Tyr(Y), Lys(K) and Arg(R) are shaded in black and their respective positions indicated. The boxes show the conserved YX_3_K motif and its corresponding PFAM HMMLogo. GenBank accession number: Eg1, EgFABG_1; Eg2, EgFABG_2; Ah, **EU823329**; At1, **AAG40337**; At2, **CAA45794**; Bn1, **CAC41362**; Bn2, **CAC41363**; Bn3, **CAC41364**; Bn4, **CAC41365**; Bn5, **CAC41370**; Ca, **ACF17653**; Cl, **CAA45866**; Os1, **ABA97197**; Os2, **BAD22913**; Ec, **ACF17653**. Abbreviations: Ah, *A. hypogaea*; At, *A. thaliana*; Bn, *B. napus*; Ca, *C. annuum*; Cl, *C. lanceolata*; Os, *O. sativa*; Ec, *E. coli*. (*) fully conserved residues; (:) conservation between groups of strongly similar properties, (.) conservation between groups of weakly similar properties.





Figure S9: Multiple sequence alignment of β-hydroxyacyl-ACP dehydrogenase (FABZ) homologues. Two catalytic dyad His(H) and Glu(E) are shaded in black and their respective positions indicated. The boxes show the conserved LPHRFPFLLVDRV domain and its corresponding PFAM HMMLogo. GenBank accession numbers: Eg1, EgFABZ_1; Eg2, EgFABZ_2; Ah, **EU823332**, At1, **AAD23619**; At2, **AAM64584**; At3, **AAO24548**; Bn, **AAK60545**; Ca, **ACF17652**; Os, **AAT58880**; Pm1, **ABA25920**; Pm2, **ABA25921**; Ec, **AAC36917**. Abbreviations: Ah, *A. hypogaea*; At, *A. thaliana*; Bn, *B. napus*; Ca, *C. annuum*; Os, *O. sativa*; Pm, *Picea mariana*; Ec, *E. coli*. (*) fully conserved residues; (:) conservation between groups of strongly similar properties, (.) conservation between groups of weakly similar properties.





Figure S10: Multiple sequence alignment of enoyl-ACP reductase (FABI) homologues. The three conserved residues Tyr(Y), Tyr(Y) and Lys(K) are shaded in black and their respective positions indicated. The boxes shows the conserved YGGGMSSAK motif and its corresponding PFAM HMMLogo. GenBank accession numbers: Eg1, EgFABI_1; Eg2, EgFABI_2; Ah, **EU823333**; At1, **AAF37208**; At2, **AAM45010**; At3, **CAA74175**; Bn1, **AAB20114**; Bn2, **CAA64729**; Bn3, **CAC41366**; Bn4, **CAC41367**; Bn5, **CAC41368**; Bn6, **CAC41369**; Oe, **AAL93621**; Ca1, **ACF17650**; Ca2, **ACF17651**; Nt1, **CAA74176**; Nt2, **CAA74177**; Os1, **BAD03622**; Os2, **BAD26009**; Os3, **CAA05816**; Ec, **P29132**. Abbreviations: Eg, *E. guineensis*; Ah*, A. hypogaea*; At, *A. thaliana*; Bn, *B. napus*; Oe, *Olea europaea*; Ca, *C. annuum*; Nt, *Nicotiana tabacum*; Os, *O. sativa*; Ec, *E. coli*. (*) fully conserved residues; (:) conservation between groups of strongly similar properties, (.) conservation between groups of weakly similar properties.





Figure S11: Multiple sequence alignment of stearoyl-ACP desaturase (FAB2) homologues. The boxes show two conserved EENRH and DEKRH motifs and their corresponding PFAM HMMLogo. The positions are indicated. GenBank accession numbers: Eg1, EgFAB2_1; Eg2, EgFAB2_2; Eg3, EgFAB2_3; Eg4, EgFAB2_4; Eg5, EgFAB2_5; Eg6, EgFAB2_6; At1, **NP_175048**; At2, **NP_181899**; At3, **NP_186910**; At6, **NP_197127.1**; Gm1, NP_001236391; Gm2, **NP_00128544**; Os1, **NP_001045215**; Os2, **NP_001046913.1**; Os3, **NP_001052594**; Os4, **NP_001061209**; Rc1, **XP_002526163.1**; Rc2, **XP_002514780**; Rc3, **XP_002531889**; Zm1, **NP_001130573**; Zm2, **NP_001131336**; Zm3, **NP_001132757**; Zm4, **NP_001140670.1**. Abbreviations: Eg, *E. guineensis*; At, *A. thaliana*; Gm, *G. max*; Os, *O. sativa*; Rc, *R. communis*; Zm, *Zea mays*. (*) fully conserved residues; (:) conservation between groups of strongly similar properties, (.) conservation between groups of weakly similar properties.





Figure S12: Multiple sequence alignment of oleoyl-phosphatidylcholine desaturase (FAD2) homologues. Eight histidine residues are shaded in black and their corresponding positions indicated. The boxes show three separate clusters of conserved regions and their corresponding PFAM HMMLogo. GenBank accession numbers: Eg1, EgFAD2_1; Eg2, EgFAD2_2; At1, **P46313.1**; At2, **AAA32782.1**; At3, **AAG51042.1**; At4, **AAM61113.1**; At5, **CAJ18799.1**; Gm1, **AAB00860.1**; Gm2, **AAB00859.1**; Gm3, **NP_001238342.1**; Gm4, **AAT44123.1**; Gm5, **AAX29989.1**; Gm6, **NP_001237865.1**. Abbreviations: Eg, *E. guineensis*; At, *A. thaliana*; Gm, *G. max*. (*) fully conserved residues; (:) conservation between groups of strongly similar properties, (.) conservation between groups of weakly similar properties.





Figure S13: Multiple sequence alignment of linoleoyl-phosphatidylcholine desaturase (FAD3) homologues. Eight histidine residues are shaded in black and their respective positions indicated. The boxes show three separate clusters of conserved regions and their corresponding PFAM HMMLogo. GenBank accession numbers: Eg1, EgFAD3_1; Eg2, EgFAD3_2; Eg3, EgFAD3_3; At1, **AEC08331.1**; At2, **NP_180559.1**; At3, **AAA61778.1**; At4, **BAA05514.1**; Gm1, **NP_001236943.1**; Gm2, **NP_001237507.1**; Gm3, **NP_001236783.1**; Gm4, **NP_001236114.1**; Gm5, **ABV00680**. Abbreviations: Eg, *E. guineensis*; At, *A. thaliana*; Gm, *G. max*, Ec. *E*. *coli*. (*) fully conserved residues; (:) conservation between groups of strongly similar properties, (.) conservation between groups of weakly similar properties.





Figure S14: Multiple sequence alignment of acyl-ACP thioesterase (FATB) homologues. Three residues comprising of the catalytic triad Asn(N), His(H) and Cys(C) are shaded in black. The boxes show the conserved active-site motifs and their corresponding PFAM HMMLogo. GenBank accession number: Eg1, EgFATB_1; Eg2, EgFATB_2; Eg3, EgFATB_3; Eg4, EgFATB_4; At, **NP_172327.1**; Gm1, **NP_001237802.1**; Gm2, **XP_003527137.1**; Gm3, **NP_001254000.1**; Gm4, **XP_03526946.1**; Os1, **NP_001057985.1**; Os2, **NP_001068400.1**; Rc1, **XP_002517525.1**; Rc2, **XP_002515564.1**; Rc3, **XP_002511148.1**; Zm1, **NP_001147887.1**; Zm2, **NP_001149963.1**; Zm3, **NP_001151014.1**; Zm4, **NP_001151366.1**. Abbreviations: Eg, *E. guineensis*; At, *A. thaliana*; Gm, *G. max*; Os, *O. sativa*; Rc, *R. communis*; Zm, *Z. mays*. (*) fully conserved residues; (:) conservation between groups of strongly similar properties, (.) conservation between groups of weakly similar properties.





Figure S15: Multiple sequence alignment of oleoyl-ACP thioesterase (FATA) homologues. Three residues comprising of the catalytic triad Asn(N), His(H) and Cys(C) are shaded in black. The boxes show the conserved active-site motifs and their corresponding PFAM HMMLogo. GenBank accession number: Eg1, EgFATA_1; Eg2, EgFATA_2; At, **NP_189147.1**; Gm1, **XR_415761.1**; Gm2, **XP_006602508**; Os, **NP_001063601.1**; Rc, **XP_002532744.1**; Zm, **NP_001130099**.1. Abbreviations: Eg, *E. guineensis*; At, *A. thaliana*; Gm, *G. max*; Os, *O. sativa*; Rc, *R. communis*; Zm, *Z. mays*. (*) fully conserved residues; (:) conservation between groups of strongly similar properties, (.) conservation between groups of weakly similar properties.





Figure S16: Phylogenetic tree of FATA and FATB. Abbreviations: Eg, *E. guineensis*; At, *A. thaliana*; Gm, *G. max*; Os, *O. sativa*; Rc, *R. communis*; Zm, *Z. mays*.





Figure S17: Genomic segmental duplication identified in FA genes. (a) *EgFABF_2* and *EgFABF_3*, (b) *EgFABH_1* and *Eg FABH_3*, (c) *EgFAD3_1* and *EgFAD3_2*. Green boxes show the location of the genes.
