# Supplementary material for: Evidence-based gene models for structural and functional annotations of the oil palm genome
Source: Biol Direct. 2017 Sep 8;12:21. doi: 10.1186/s13062-017-0191-4 (PMC5591544; doi:10.1186/s13062-017-0191-4)
Supplement: Supplementary file 4 — Additional file 4 provides screenshots of PalmXplore. (DOCX 962 kb) [file 13062_2017_191_MOESM4_ESM.docx]

Additional file 4


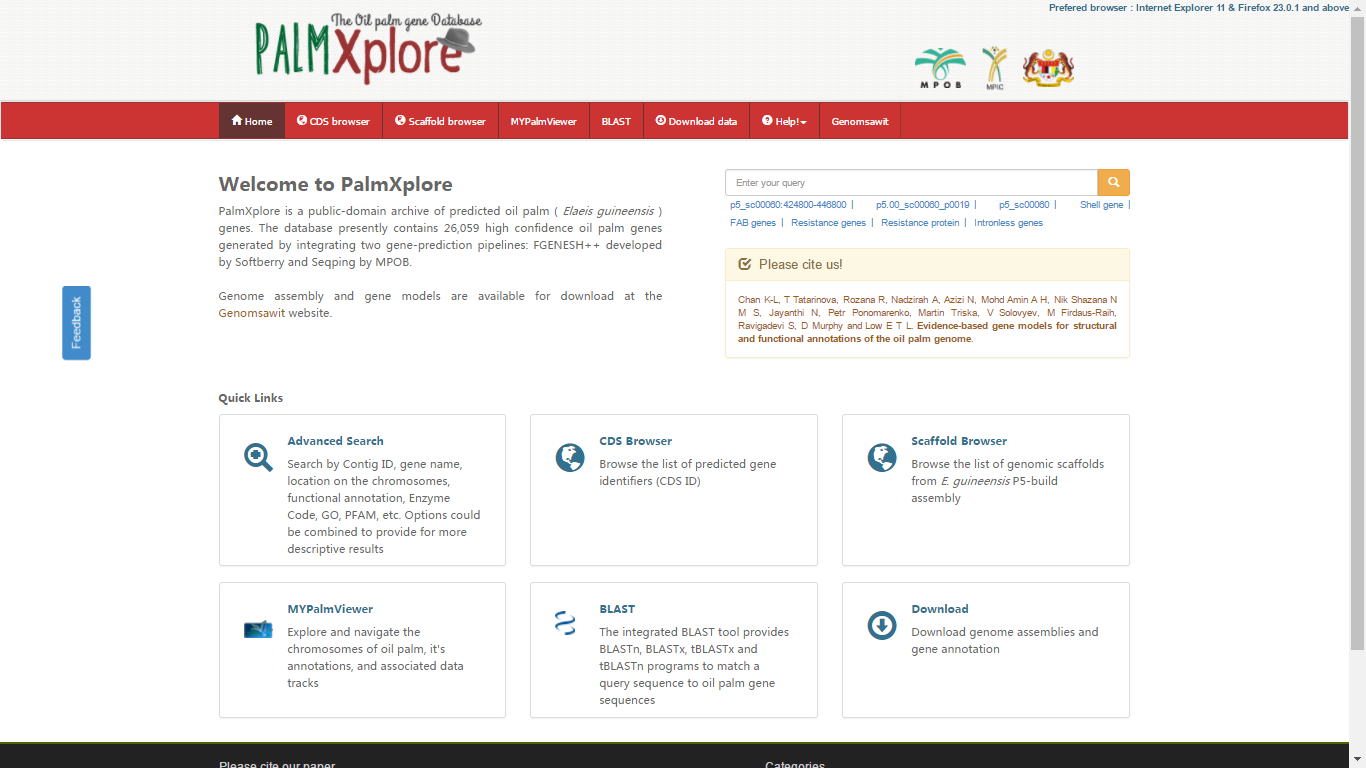


**A**

Figure S18: PalmXplore home page and basic search. (A) Basic Search: Search oil palm genes by Gene ID, Scaffold ID, functional annotation keyword, specific gene or location on the genome


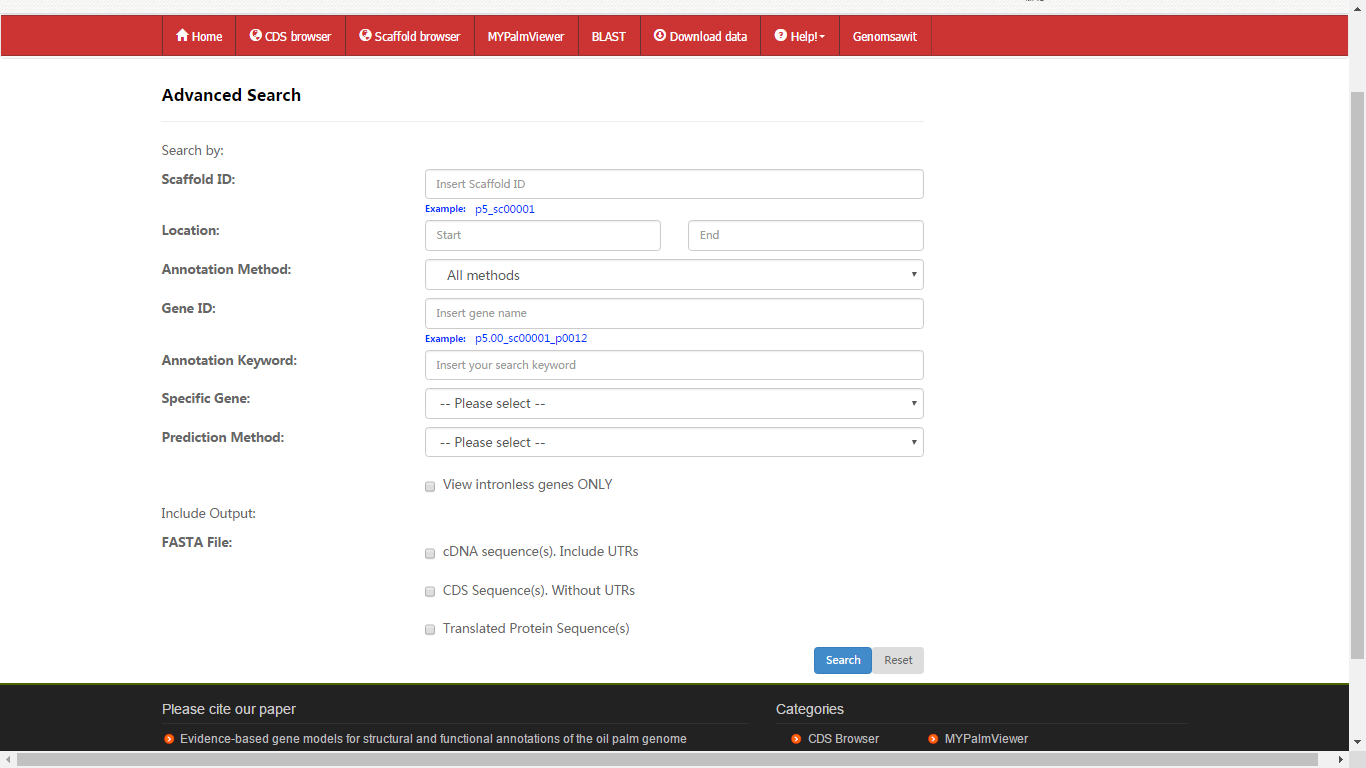


Figure S19: Advanced search. Refine search results by entering multiple options


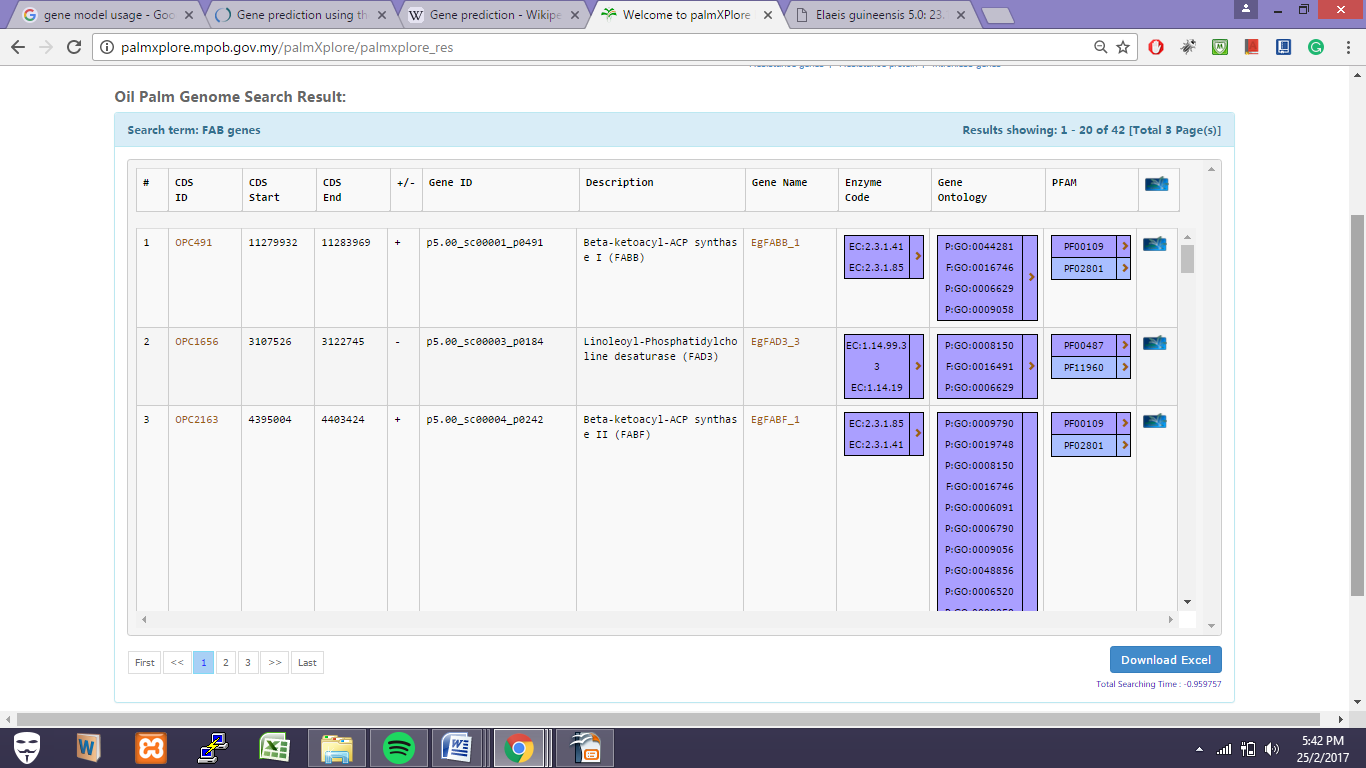


Figure S20: Search results. Search results shows links to CDS information, and links to gene location on MYPalmViewer genome browser and external online databases


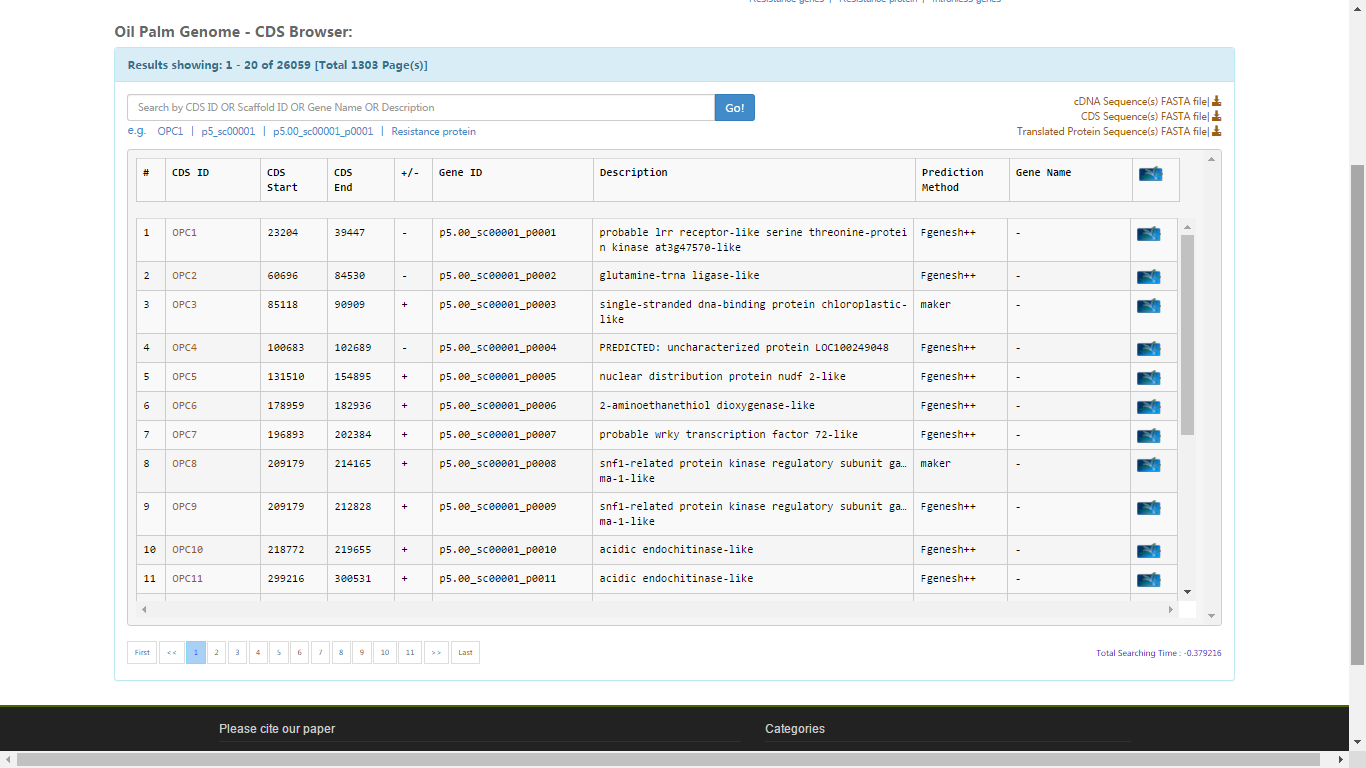


Figure S21: CDS browser


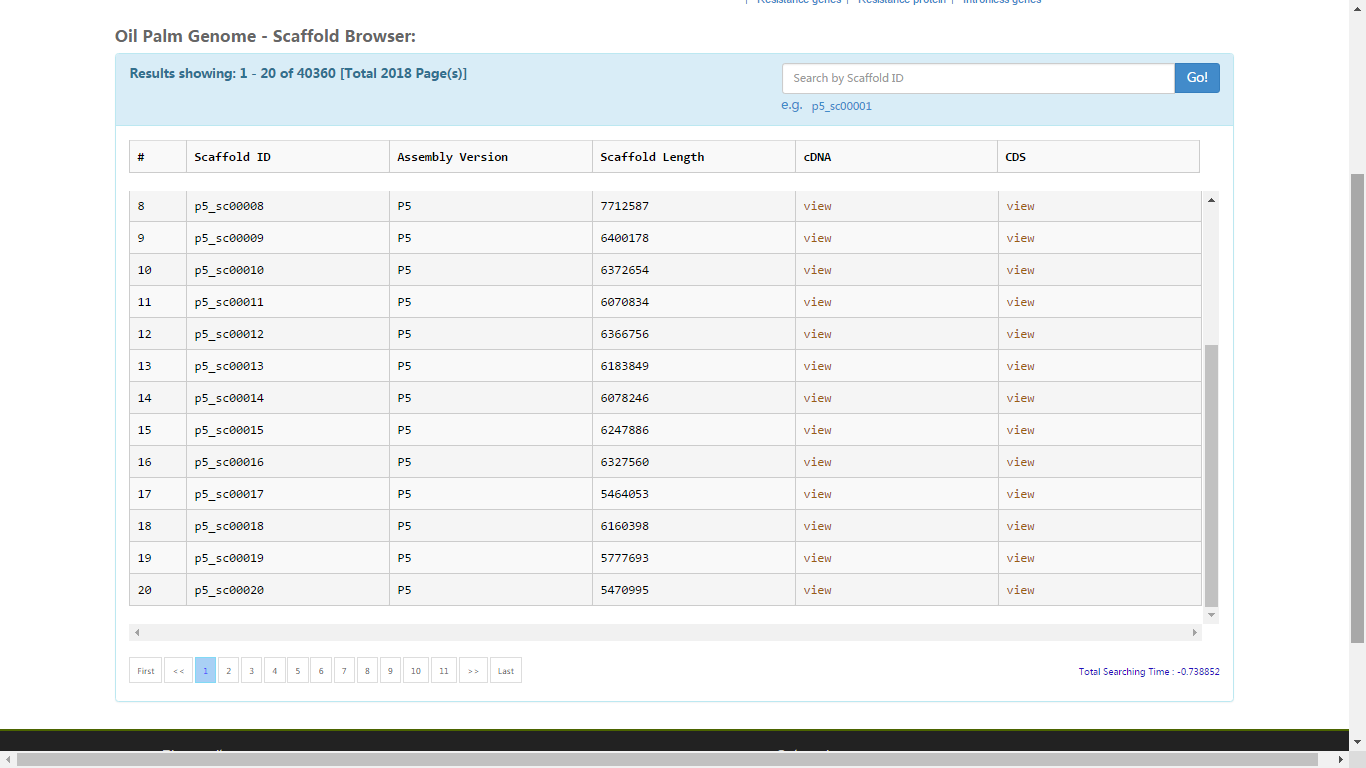


Figure S22: Scaffold browser


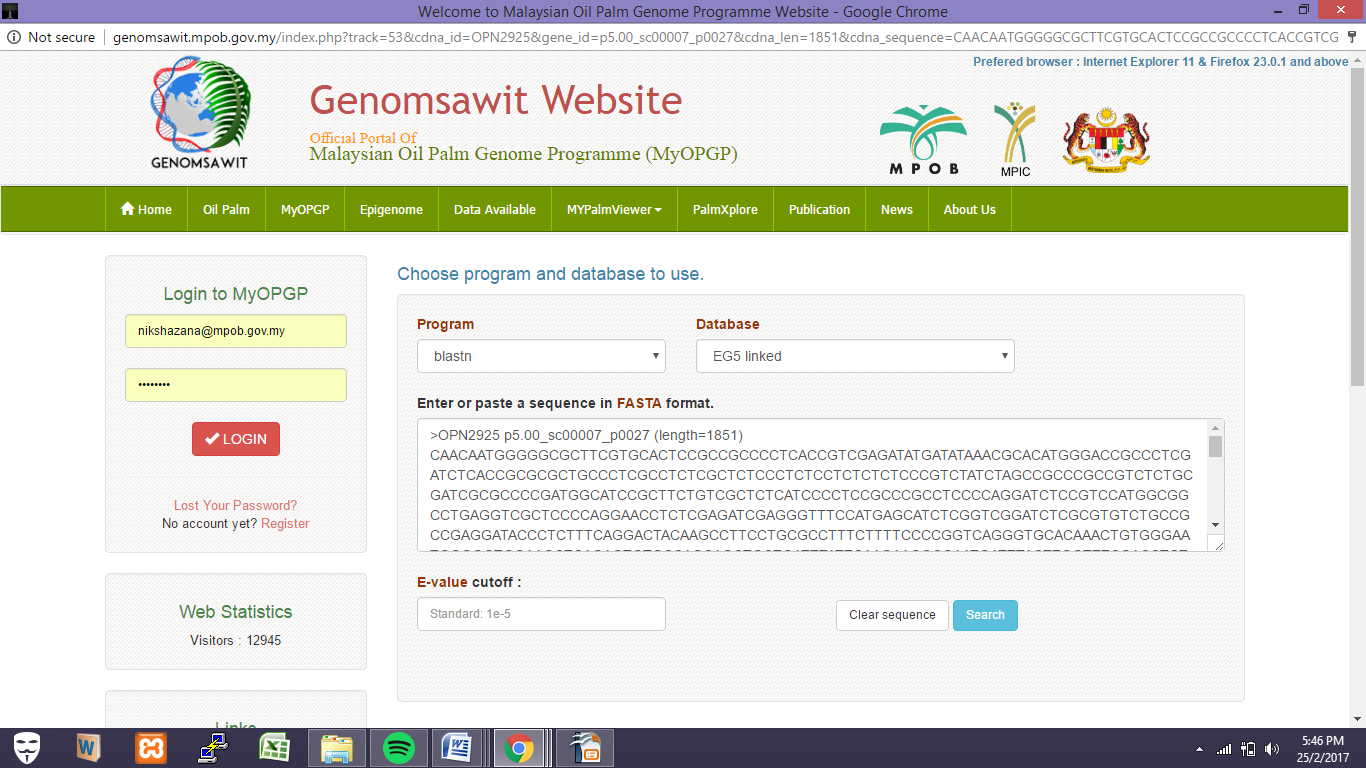


Figure S23: BLAST tool. The gene sequence of interest is auto-filled into the text-field


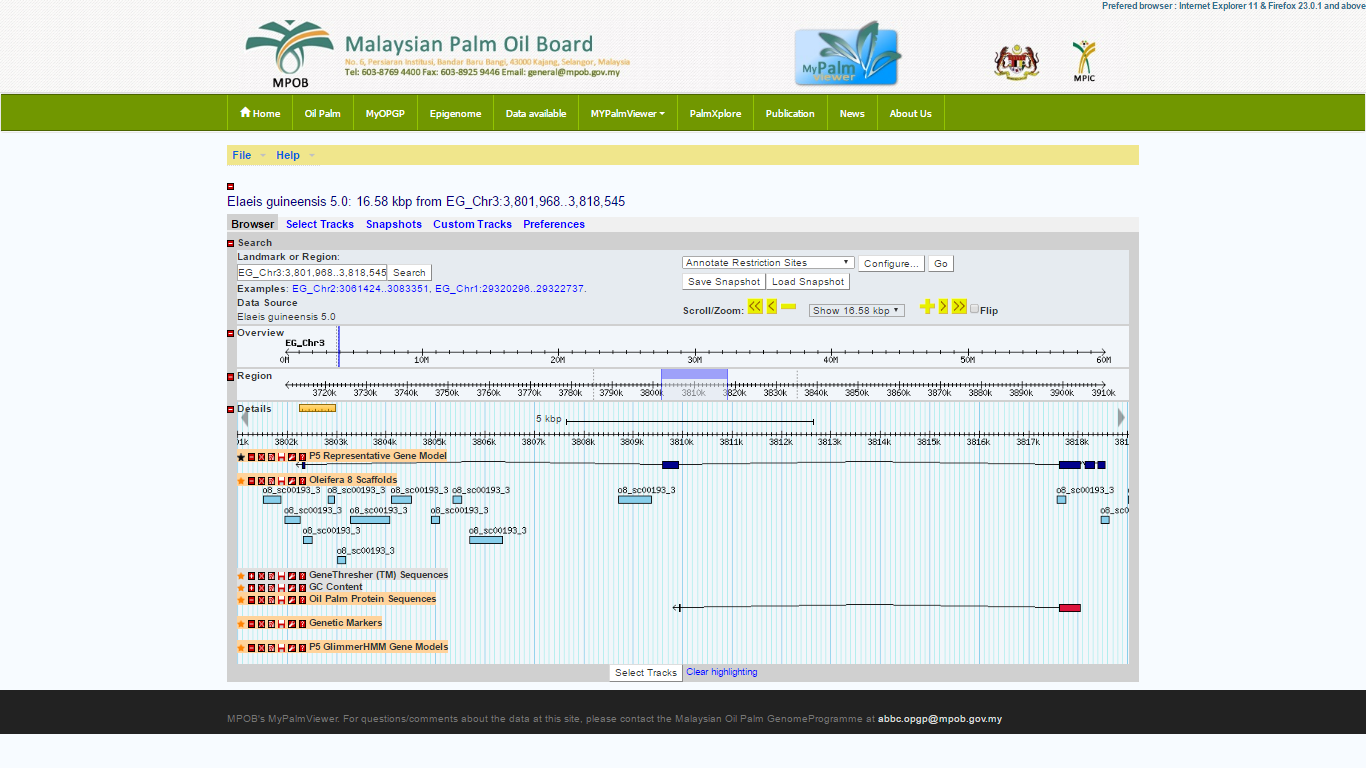


Figure S24: MYPalmViewer oil palm genome browser
